# Computerized tomography image correlation of His bundle/deep septal pacing location and outcomes: an analysis from the Canberra HIs bundle/deep septal Pacing Study (CHIPS)

**DOI:** 10.1007/s10840-022-01133-z

**Published:** 2022-01-27

**Authors:** Sreevilasam P. Abhilash, Deep Chandh Raja, Simon Stolcman, Dong Seok YI, Moyazur Rahman, Ren Tan, Aakash Mahajan, Dennis H. Lau, Walter P. Abhayaratna, Prashanthan Sanders, Rajeev Kumar Pathak

**Affiliations:** 1 Canberra Heart Rhythm, Australian National University, Canberra, ACT Australia; 2 Cardiac Electrophysiology Unit, Department of Cardiology, Canberra Hospital, Yamba Drive, Garran, ACT 2605 Australia; 3 Centre for Heart Rhythm Disorders, South Australian Health and Medical Research Institute, Adelaide, Australia

**Keywords:** His bundle pacing (HBP), Para-Hisian pacing, Left bundle branch pacing (LBBP), Deep septal pacing

## Abstract

**Background:**

Localisation of the conduction system under fluoroscopy is not easy and the ideal location of the pacing leads in physiological pacing is still being debated.

**Objective:**

The primary aim was to assess the lead locations using cardiac CT scan. Secondary aims were clinical outcomes including success and safety of the procedure and lead performance.

**Methods:**

Of the 100 consecutive patients who received physiological pacing, 34 patients underwent follow-up cardiac CT scan. The four different types of pacing were identified as His bundle (HBP), para-Hisian, left bundle branch (LBBP), and deep septal pacing.

**Results:**

Most patients had successful HBP via the right atrium (RA) (87.5%) as compared to the right ventricle (RV) (12.5%). Lower thresholds were observed when leads were placed within 2 mm of the junction of the membranous and muscular ventricular septum. Unlike HBP, LBBP was possible at a wide region of the septum and selective capture of individual fascicles was feasible. LBBP showed deeper penetration of leads into the septum, as compared to deep septal pacing (70% vs. 45%). Approximately, 80% of patients did not have an intra-ventricular portion of the membranous septum.

**Conclusions:**

The anterior part of the atrio-ventricular (AV) septum at the junction between the membranous and muscular septum via RA appeared to be the best target to successfully pace His bundle. LBBP was possible at a wide region of the septum and selective capture of individual fascicle was feasible. Adequate depth of penetration of lead was very important to capture the left bundle.

**Supplementary Information:**

The online version contains supplementary material available at 10.1007/s10840-022-01133-z.

## Introduction

Conventional right ventricular pacing is known to cause ventricular dyssynchrony, heart failure, and increased mortality. By maintaining conduction through the normal His-Purkinje system, ‘physiological pacing’ prevents ventricular dyssynchrony and may prevent pacing-mediated cardiomyopathy [1]. His bundle pacing (HBP) was described initially as the ‘most physiological form of pacing’ since the pacing lead captures the His bundle directly and subsequent travel of impulses occurs through the conduction system resulting in a narrow QRS and physiologic activation of the ventricular myocardium [2, 3]. Of late, left bundle branch pacing (LBBP) [4, 5] has been described where pacing across the inter-ventricular septum can capture the left bundle thus resulting in pacing distally through the conduction system [6–8].

**Fig. 1:**
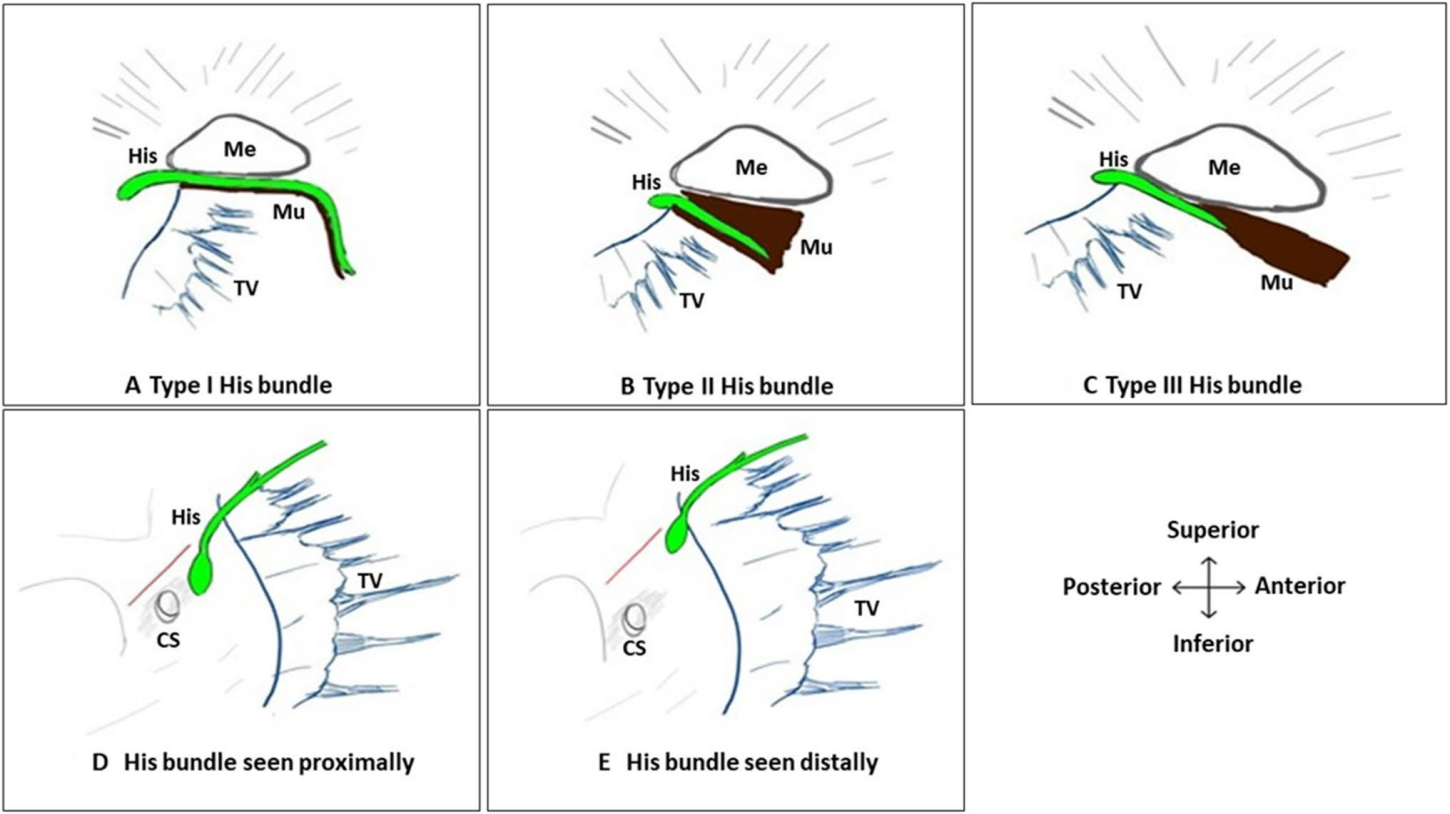
The Schematic diagram of relevant anatomy. Conduction system (green colour) with respect to the membranous septum (Me-triangular area), the upper end of the muscular septum (Mu -brown shaded) and septal leaflet of the tricuspid valve (TV-blue colour). **A–C** show muscular septum and membranous septum in the sagittal section depicting the His bundle in superior to the inferior plane; the three types of His bundles are shown. **D and E** show compact AV node and His bundle (green colour) in relation to Koch’s triangle as viewed from RA. The red line is the tendon of Todaro. Septal attachment of tricuspid valve (TV-blue colour) and coronary sinus (CS) opening also shown. In **E**, please note that the compact AV node and the His bundle are placed further anterior (distal) and superior to the CS mouth compared to **D**

Kawashima et al. described three distinct types of His bundle that were all described close to the junction between the membranous and muscular septum [9]. This study provided insights into the vertical (superior-inferior) orientation of the conduction system axis with respect to the junction of the membranous and muscular septum. Figure 1A shows type 1 His bundle which lies at the junction between the membranous and muscular septum. Figure 1B shows type 2 His bundle within the muscular septum but His bundle is seen closer to the membranous septum near the tricuspid valve (TV) attachment. In type 3 (Fig. 1C), His bundle is ‘naked’ and seen immediately below the membranous part of the interventricular septum.

Additionally, the study by Cabrera et al. showed a marked variation of the location of the transition from the atrioventricular (AV) conduction axis to the bundle of His, relative to the landmarks of the triangle of Koch [10]. This study revealed the horizontal (antero-posterior) alignment of the axis in relation to the septal leaflet of TV. In up to half of the patients, the site of penetration was closer to the atrial aspect of the hinge of the septal leaflet of TV (Fig. 1D–E). His bundle is seen anteriorly near the TV attachment (Fig. 1D) in half of the patients and further distally (Fig. 1E) in the remaining half (Supplementary appendix 2).

Despite the potential advantages of the physiological pacing, the localisation of His bundle/LBB in real world under fluoroscopy is challenging [11, 12]. Apart from case reports [13, 14], there is only one study [15] which has specifically investigated the location of the pacing leads by cardiac imaging in relation to the type of pacing. The present study primarily aimed to look at the pacing lead locations at the follow-up cardiac computerised tomography (CT) scan and correlate with the conduction system capture on the 12-lead electrocardiograms (ECG). Secondary aims were assessment of the clinical outcomes including success and safety of the procedure and lead performance at the insertion and follow-up.

## Methods

### Study population

A total of 100 patients received the physiological pacing between September 2017 and August 2020 at Canberra hospital, Australia. Of these, 37 patients underwent cardiac CT scans post lead insertion to rule out coronary artery disease. In 3 patients, the CT scans had to be excluded from analysis due to lead artefacts or poor-quality images. The remaining 34 patients were included in this study. The study was approved by the Canberra hospital ethics and governance committee.

#### Implantation procedure

Mapping of the His bundle was done with the Select Secure 3830 lead (Medtronic, Minneapolis, MN) through the C315 His fixed curve delivery sheath. Mapping was performed using the standard fluoroscopic views and recording of a His bundle ECG was attempted in all the cases. If the His bundle potential was difficult to record or a His capture was not obtained despite multiple attempts, the lead was screwed in at a location based on the surface ECG where passive pacing suggested a para-Hisian location. As the evidence for LBBP started accumulating, we started performing LBBP more because of the potential advantages over HBP [6, 7]. Every effort was made to record a LBB potential after screwing in the lead to the LBB location. If an LBB potential was not recorded or the LBB capture could not be obtained after screwing deep into the septum despite > 3 attempts, the deep septal location of the screw-in lead was accepted. (See supplementary appendix 1 for details of implantation and selection of study groups.)

**Fig. 2:**
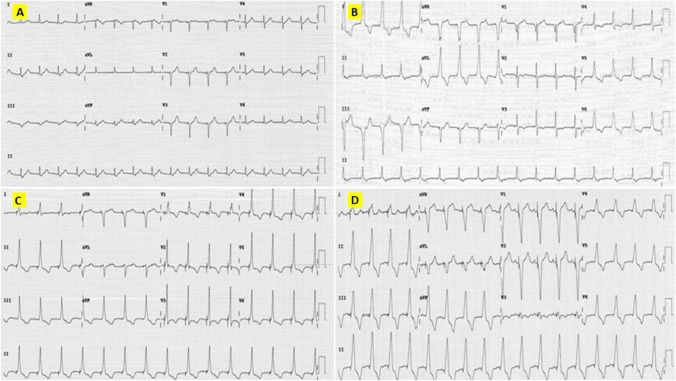
Four types of pacing observed in surface ECG. (**S**ee Appendix 3 f**or larger images**). **A** His bundle pacing—Note ‘normal looking’ narrow QRS, normal QRS axis and retrograde P waves. **B** Para-Hisian pacing: Relatively broader QRS with lead II positive and lead III negative QRS. **C** Left bundle branch pacing: Narrow QRS with qR in V1, normal QRS axis. **D** Deep septal pacing: Note broader QRS and absence of lead II positivity and lead III negativity in comparison to para-Hisian pacing

Four different types of pacing were identified based on the 12-lead surface ECGs (Fig. 2). The ECGs were done with the pacing output kept within the nominal range: 1 V to 4 V at 1 ms pulse width for the HBP and para-Hisian pacing, 1 V to 4 V at 0.4 ms pulse width for the LBBP and deep septal pacing.

1. *HBP group*: The patients whose 12 lead ECG showed a His bundle capture. Both selective and non-selective HBP were included in this group.
2. *Para-Hisian pacing group*: The patients without the His bundle capture but showed a positive paced QRS in lead II and a negative QRS in lead III suggestive of a para-Hisian location.
3. *LBBP group*: Deep septal paced patients who showed a LBB capture. Those patients with the LBBP were further analysed for a selective fascicular capture (left anterior fascicle (LAF)/left posterior fascicle (LPF)) as against the main left bundle capture.
4. *Deep septal pacing group*: Deep septal paced patients who did not demonstrate a LBB capture.

##### Cardiac CT assessment

The patients who underwent cardiac CT scans in the follow-up period were selected for the analysis of the location of the pacing leads. Best diastolic images were chosen for interpretation. The CT images were interpreted using radiant DICOM viewer software (Medixant Maciej Frankiewicz, Poland) initially and subsequently, the images were merged with the CARTOSEG CT segmentation module kit (Biosense Webster, Division of Johnson and Johnson CARTO version 6, USA) for a detailed 3D image reconstruction. The CT scans of the patients in each of the 4 study groups were analysed for the location of the ventricular pacing lead and the measurements were done in multi-planar reconstruction images to ensure accuracy and comparability across the patients. The measurements (Table 1, Appendix 2 for details of CT measurements) were done by two investigators and an average was taken for the analysis.

**Table 1:** Major CT measurements

Type of pacing	Number of CTs reviewed	Location of lead (Chamber)	Distance from pacing lead tip to insertion of the septal leaflet of TV to septum -Mean ± SD(mm)	Distance from pacing lead tip to the junction of membranous part and muscular part of septum- Mean ± SD (mm)	Distance from pacing lead tip to NCC- Mean ± SD (mm)	Distance from pacing lead tip to RCC- Mean ± SD (mm)	Depth of penetration of lead into septum (%)
His bundle pacing	14	RA	2.2 ± 1.7	0.7 ± 0.9	10.1 ± 3.3	9.5 ± 4.9	Difficult to measure
	2	RV	2.6 ± 0.3	5.5 ± 1.2	19.8 ± 5.2	13.5 ± 1.3	42 ± 1.7
Para-Hisian pacing	2	RA	13.5 ± 14.7	12.7 ± 8.6	12.9 ± 12.7	15.6 ± 5.5	51
	1	RV	2.2	4.6	10.5	18.3	48
Left bundle branch pacing	11	RV	22.8 ± 10.7	23.8 ± 10.6	32.8 ± 8.7	25.4 ± 7.1	68 ± 18.6
Deep septal pacing	4	RV	25 ± 2.2	21 ± 2.8	25.6 ± 5.9	21.1 ± 8.4	46 ± 4.4

#### Statistical methods

Statistical analyses were performed using SPSS (version 22.0, SPSS Inc, Chicago, IL). Nominal data were presented as frequencies and percentages and were compared by the chi-square test. Continuous data were presented as the mean ± standard deviation and compared with 2-tailed Student t-tests. A one-way ANOVA test was done, controlling for the co-variables. *P* value of less than 0.05 was considered significant.

## Results

Thirty-seven patients underwent a cardiac CT scan to rule out coronary artery disease during follow-up. In 3 patients, the CT scans had to be excluded from analysis due to lead artefacts or poor-quality images. The remaining thirty-four patients were included in this study. All patients received the Medtronic 3830 lead. The mean follow-up of the study was 24 ± 9 months (median of 23.5 months). The mean age of the study subjects was 67.5 ± 12.9 years and 65% of patients were males.

Eighteen patients (53%) had sick sinus syndrome, 10 patients (30%) had varying levels of AV blocks, and 6 patients (17%) received physiological pacing as part of the cardiac resynchronisation therapy (CRT) for heart failure. Of the 34 patients, 16 (47.1%) had HBP, 3 (8.8%) had para-Hisian pacing, 11 (32.4%) had LBBP, and 4 (11.8%) had deep septal pacing (Table 1).

## CT scan analysis

**Fig. 3:**
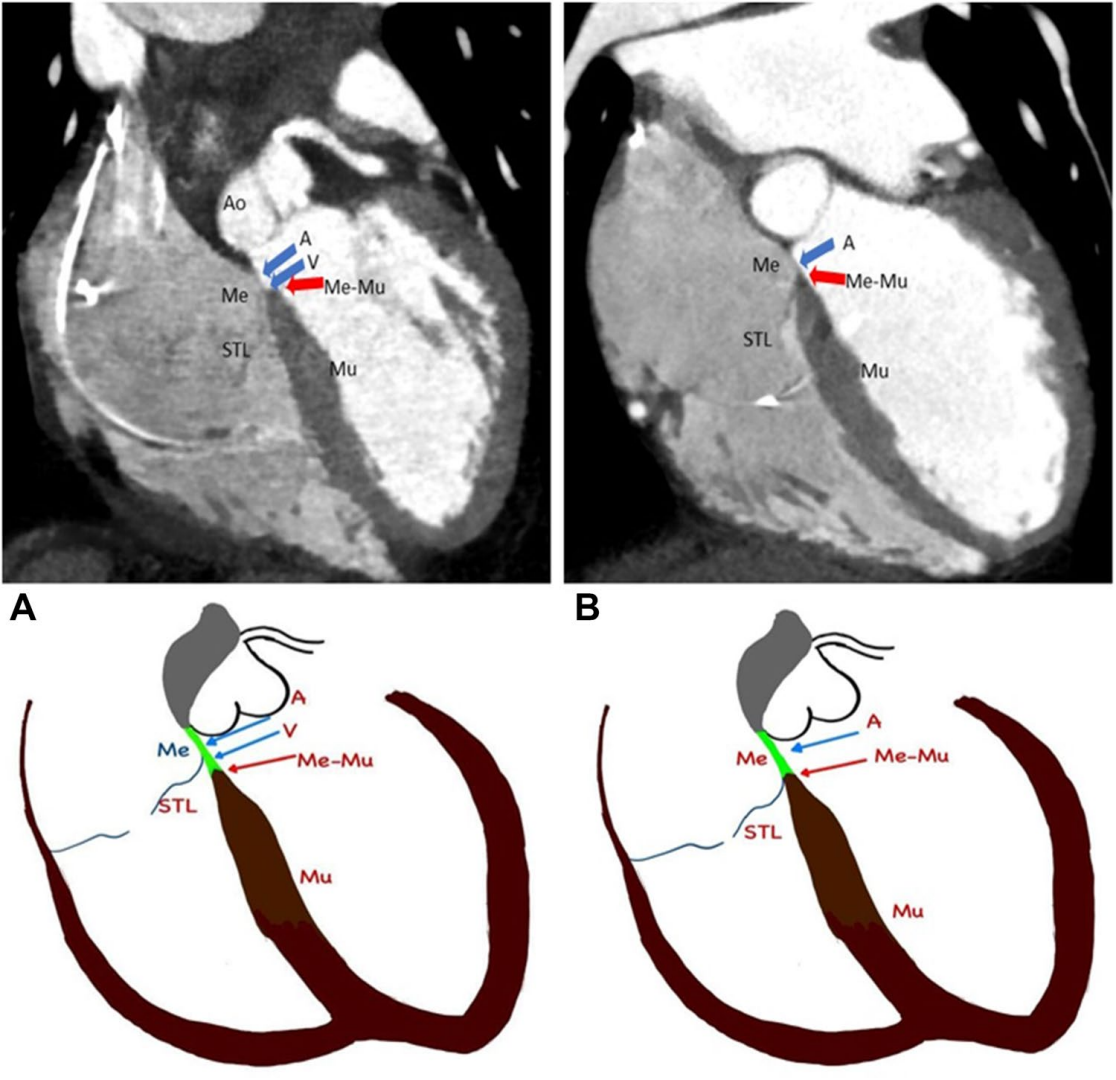
Relevant anatomy as seen in CT scan (see Appendix 3 for larger images). **A** Shows various components of the septum and relation to the septal leaflet of the tricuspid valve (STL). Me (blue arrows): membranous septum, Mu: muscular septum, A: atrial component of the membranous septum, V: ventricular component of the membranous septum, Ao: aorta, Me-Mu (red arrow): Junction between the membranous and muscular part of the septum. Corresponding schematic diagram of the CT image is given below. Please note that Me-Mu lies below the insertion of STL to the septum giving rise to the ventricular component of the membranous septum. **B** shows an absent ventricular portion of the membranous septum

The left anterior oblique (LAO) cranial view was found to be the best view to profile the septum and this view was used for the measurements. Only 7 out of 34 patients (20.6%) demonstrated a ventricular component of the membranous septum (Fig. 3).

## CT analysis of His bundle pacing

**Fig. 4:**
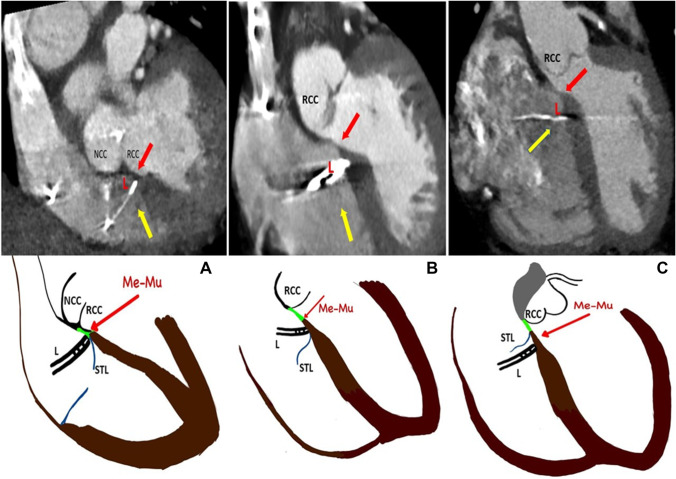
Location of the lead in His bundle pacing. (See Appendix 3 for larger images.) **A** shows HBP via RA with lead tip seen anteriorly near the right coronary cusp of the aortic valve (RCC) and away from the non-coronary cusp (NCC). Please note lead tip relationship with the Me-Mu junction (red arrow), septal leaflet of the tricuspid valve (STL-yellow arrow) and lead (L). Corresponding schematic diagram of the CT image is given below. In the schematic diagram, the membranous septum is coloured green and the muscular septum is coloured brown. Lead tip is seen at Me-Mu junction in this case. **B** shows HBP via RA who had lead tip beyond 2 mm from the Me-Mu junction. This patient had increased thresholds resulting in lead replacement on follow up. **C** shows HBP via RV. Lead tip is seen capturing muscular septum distally

Of the 16 patients demonstrating HBP on the surface ECG, the lead was found to be capturing His Bundle via RA in 14 patients (87.5%) and two patients (12.5%) had His bundle capture via RV (Fig. 4A–C, Tables 1 and 2). The distance between the lead tip to the junction between the membranous and muscular septum was 0.9 ± 0.7 mm in case of His capture via RA whereas it was 5.5 ± 1.2 mm in the case of His capture via RV. In all cases, the lead tip was seen anteriorly in the septum near the right coronary cusp (RCC) of the aorta and away from the non-coronary cusp (NCC).

**Table 2:** Threshold of pacing and distance from the junction of membranous septum and muscular septum in patients who underwent CT scan following HBP and para-Hisian pacing

Type of pacing	Serial number of patients	Lead tip within 2 mm of Me-Mu septum junction – 12 patients (Threshold—V at 1 ms)		Lead tip more than 2 mm from Me-Mu septum junction-7 patients (threshold—V at 1 ms)		Outcome on follow up
		Initial	Follow up	Initial	Follow up	
HBP via RA	1	1.0	1.0			All patients had stable parameters
	2	0.5	0.5			
	3	0.5	1.3			
	4	0.5	0.8			
	5	0.5	0.8			
	6	0.5	0.5			
	7	0.5	0.8			
	8	0.5	0.5			
	9	0.5	1.5			
	10	0.5	1.0			
	11	0.5	0.8			
	12	0.8	0.8			
HBP via RA	13			0.5	0.5	One patient required lead change
	14			1.3	4.0	
HBP via RV	1			0.5	0.5	Both patients had stable parameters
	2			0.5	0.8	
Para-Hisian pacing	1			2.0	2.0	One patient required lead change
	2			1.0	5.0	
Via RA						
Para-Hisian pacing	1			0.8	1.5	Patient remained stable
Via RV						
Mean ± SD		0.6 ± 0.7	0.8 ± 0.3	0.9 ± 0.6	2.0 ± 1.8	*P* value of 0.04 and 0.03 respectively for initial and follow up between two groups

## CT analysis of para-Hisian pacing

**Fig. 5:**
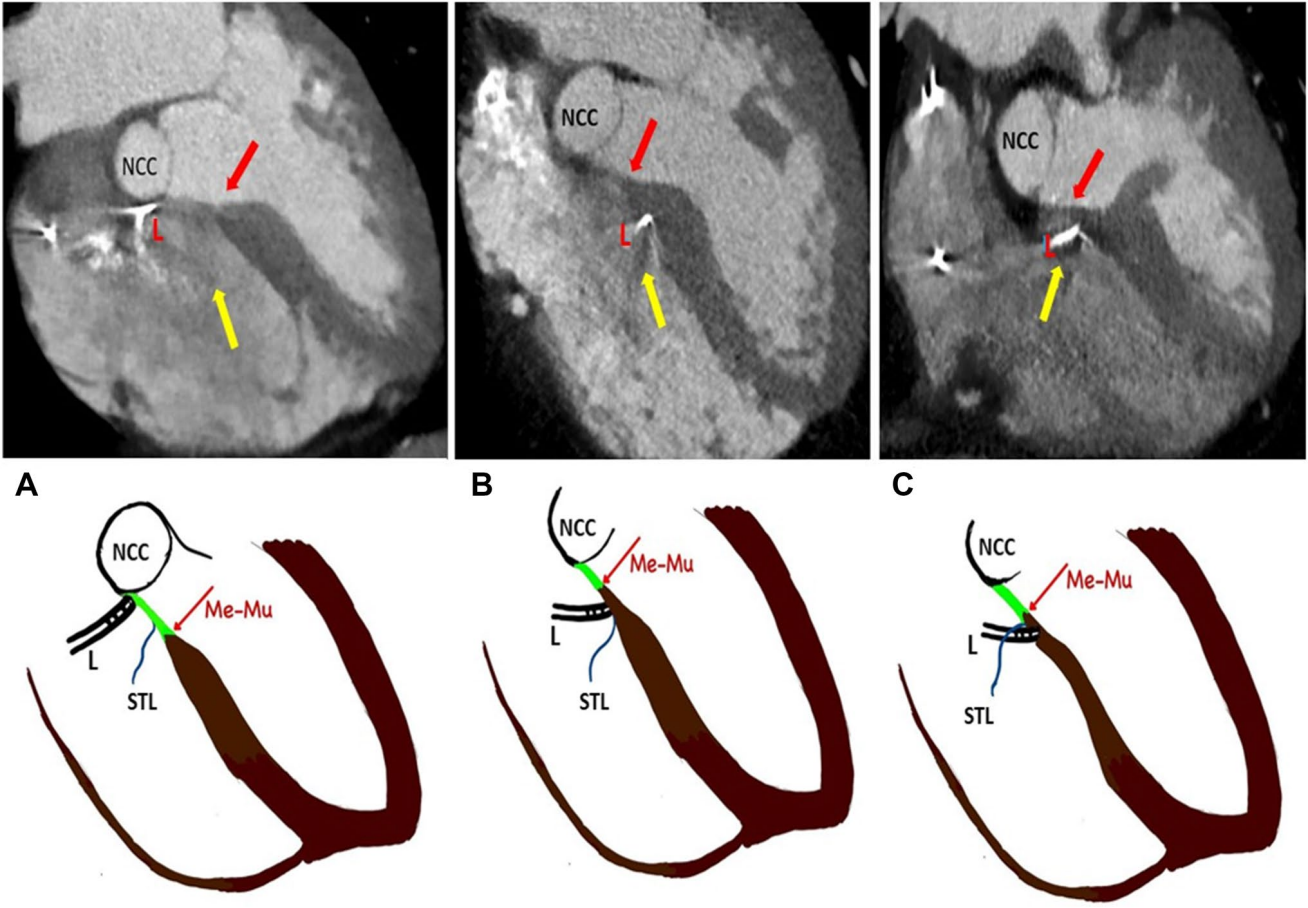
Location of the lead in para-Hisian pacing. (See Appendix 3 for larger images)**. A** shows para-Hisian pacing via RA and the lead is seen posteriorly near non-coronary cusp of the aorta (NCC). Please note that the lead tip is much posterior and away from the Me-Mu junction. Me-Mu junction (red arrow), septal leaflet of tricuspid valve (STL -yellow arrow) and lead (L). Corresponding schematic diagram of CT image is given below. In the schematic diagram, the membranous septum is coloured green and the muscular septum is coloured brown. **B** shows para-Hisian pacing via RA and the lead tip is located beyond 2 mm from the Me-Mu junction. Note ‘atrial component’ of muscular septum. This patient had increased thresholds and required lead revision. Since the lead is not normally screwed deeply in HBP, the thick muscular septum might have led to high thresholds to capture the conduction system. **C** shows para-Hisian pacing via RV. Note that the lead tip is seen below the TV attachment into the RV

The para-Hisian pacing was via RA in 2 patients and via RV in 1 patient (Fig. 5A–C Tables 1, 2). The first patient with para-Hisian pacing via RA had the lead screwed in posteriorly in the membranous septum near NCC (Fig. 5A). Since the lead was screwed in posteriorly, lead tip was 18.8 mm away from the junction between the membranous and muscular septum and 23.9 mm away from the septal leaflet of TV on the atrial side. At baseline, the threshold was 2 V at 1 ms pulse width and R wave was 1 mV; however, this remained the same at 29 months of follow-up.

The second patient with para-Hisian pacing via RA had the pacing lead screwed in anteriorly to the AV septum (Fig. 5B). In this patient, the muscular septum was seen above the septal leaflet of TV into RA and the lead was screwed into this ‘atrial component of the muscular septum.’ The lead was found to be 6.7 mm away from the junction between the membranous and muscular septum and 3.1 mm away from the septal leaflet of TV. The initial R wave was poor at 1 mV, but the threshold was acceptable at 1 V at 1 ms. However, the threshold worsened, and the patient required a lead repositioning at 8 months follow-up.

The third patient with para-Hisian pacing had the lead 2.2 mm below the TV attachment into the RV side of the muscular septum, 4.6 mm away from the junction between the membranous and muscular septum. (Fig. 5C). R wave was 1.9 mV and the threshold was 0.75 V at 1 ms pulse width which remained at 1.5 V at 1 ms pulse width at 22 months of follow-up.

## CT analysis of left bundle branch pacing

**Fig. 6:**
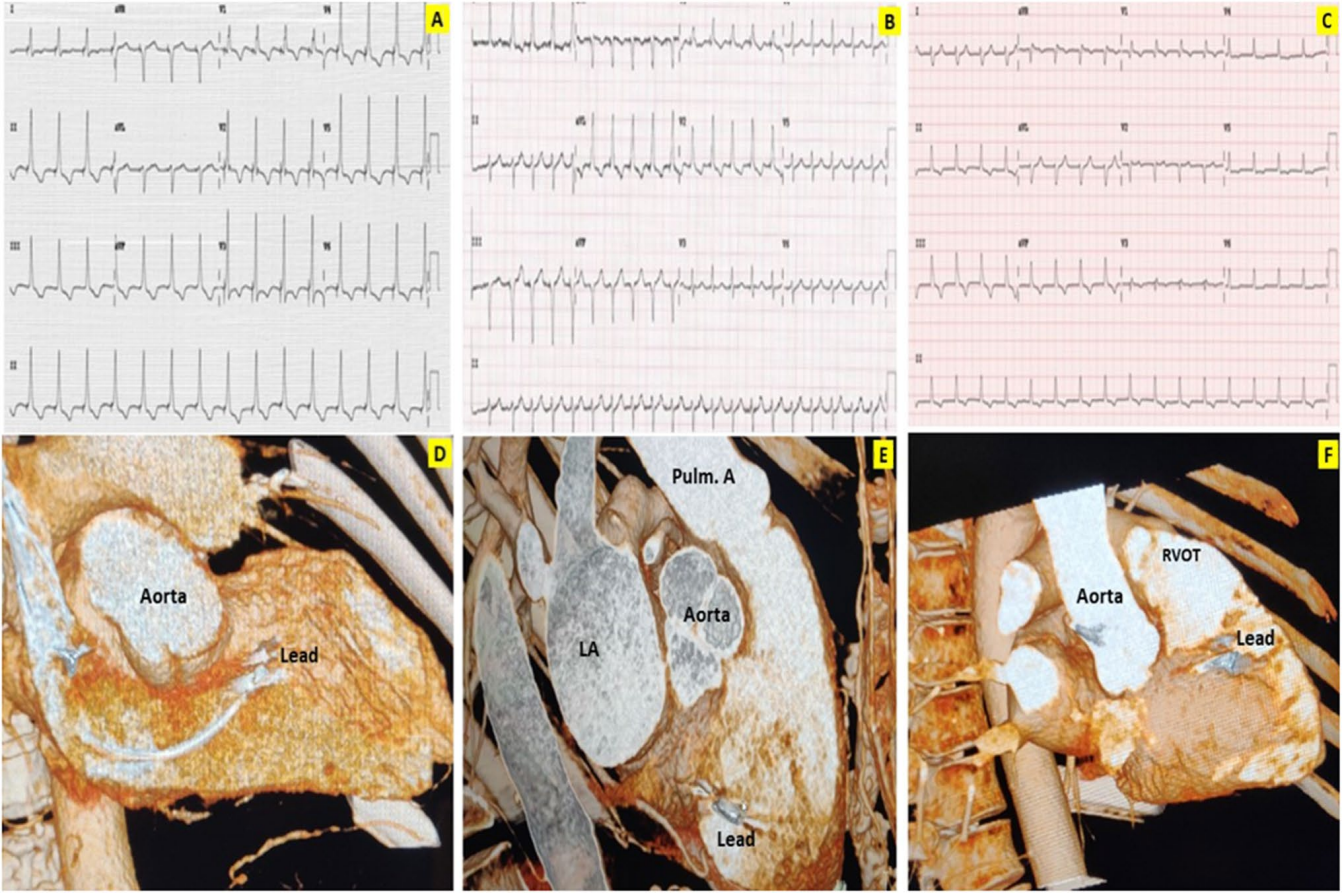
Left bundle branch pacing. **A** shows main left bundle capture, **B** shows left posterior fascicle (LPF) capture, **C** shows left anterior fascicle (LAF) capture. (See Appendix 3 for larger images). Note narrow QRS and qR patterns in V1 in all ECGs. **A** shows normal QRS axis suggesting main left bundle capture. **B** shows the left axis deviation of QRS and **C** shows the right axis deviation of QRS suggesting capture of LPF and LAF respectively. **D–F** show corresponding CT images of the pacing lead location confirming existing understanding of the location of fascicles of the left bundle

There were 11 patients with LBBP whose CT scans were available for analysis. (Table 1). The depth of penetration of the lead into the septum was 68 ± 18.6%. The mean distance from the lead tip to TV insertion was 22.8 mm (Fig. 6A, D). Three patients (27.3%) had LPF capture at all pacing outputs and the lead was noted in the posterior third of the interventricular septum (Fig. 6 B, E). Two patients (18.2%) had LAF capture (Fig. 6 C, F) with the pacing lead located at the anterior third of the interventricular septum towards outflow.

## CT analysis of deep septal pacing

**Fig. 7:**
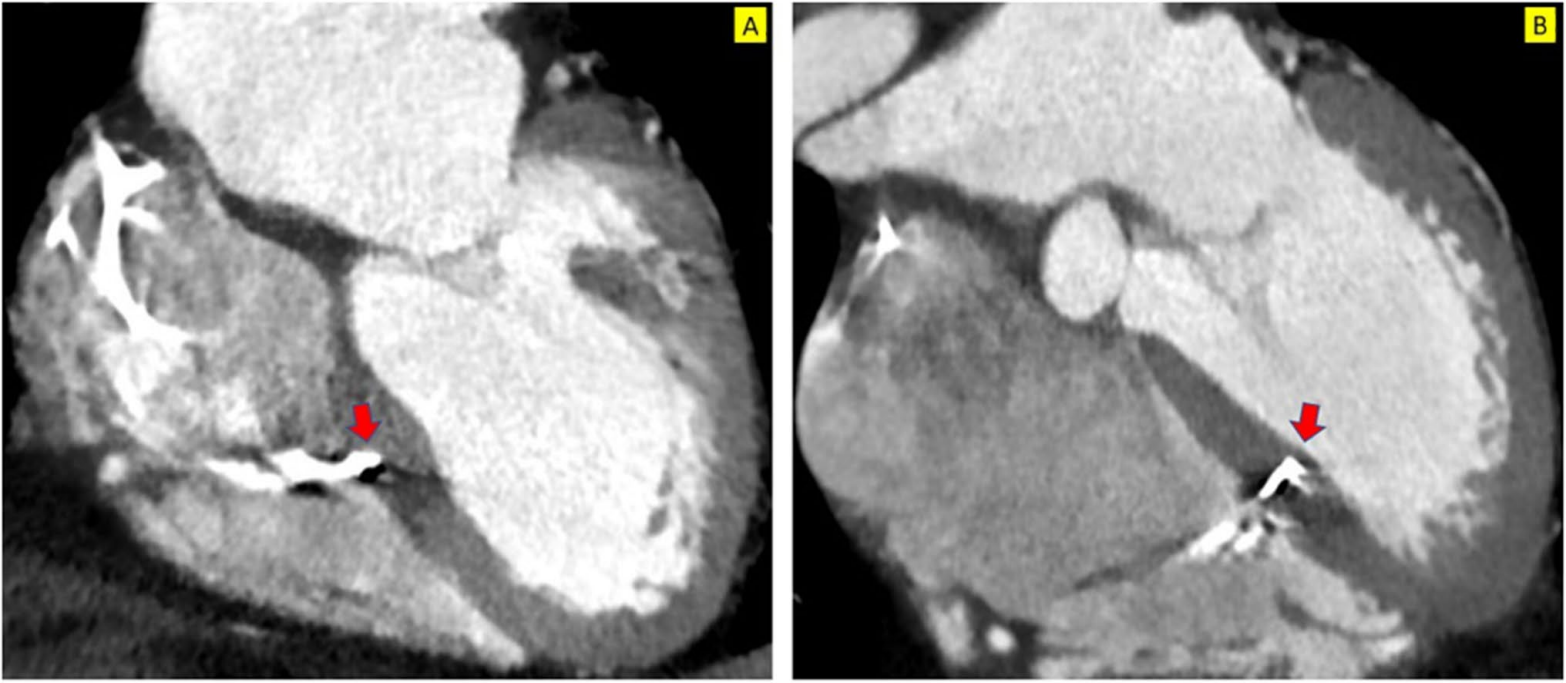
Depth of penetration of lead into the septum. **A** shows a case of deep septal pacing. LBBP is shown in **B**. Deep septal pacing lead has penetrated only up to 30% of depth into septum whereas LBBP lead tip is seen at 80% depth. Red arrow: lead tip

The analysis of CT scans was available for 4 patients with deep septal pacing (Table 1). The depth of penetration of the lead into the septum at 46 ± 4.4% was observed to be significantly less than LBBP. (68 ± 18.6 vs 46 ± 4.4, p = 0.04) (Fig. 7 A, B). The mean distance from the tip of lead to the TV insertion was 25 mm.

## Clinical outcomes

### Lead threshold

Overall, HBP and para-Hisian pacing had comparable thresholds (Tables 1 and 2). About 85% of patients with HBP had the lead within 2 mm of the junction between the membranous and muscular septum in the RA. These patients had lower thresholds at the time of implantation (0.77 ± 0.6 V, *P* value 0.04) and follow up (1.56 ± 1.7 V, *P* = 0.03). Among all the cohorts, LBBP had the lowest threshold with the mean threshold of 0.6 ± 0.3 V at 0.4 ms pulse width during the implantation and 0.85 ± 0.4 V at final follow-up. However, this was not statistically different when compared to the threshold of deep septal pacing at the implantation or follow-up.

## ECG characteristics

HBP and LBBP resulted in significantly reduced QRS duration when compared to para-Hisian pacing and deep septal pacing, respectively. Of note, a ventricular capture was seen in the para-Hisian pacing group despite a CT scan demonstrating the location of the ventricular lead to be in RA in two of three patients. The lead tips in RA were noted to be at the AV septum, suggesting that the ventricle could be captured from the AV septum via RA. Among patients who underwent LBBP, 3 patients (27.3%) had LPF, and 2 patients (18.2%) had LAF capture.

## Complications

Of the 34 patients in this study, two patients required lead revisions. The first patient had HBP, with the lead tip more than 2 mm away from the junction of the membranous and muscular septum (Fig. 4B). The second patient had para-Hisian pacing with the lead tip seen 6.4 mm away from the junction of the membranous and muscular septum (Fig. 5B).

## Discussion

This study gives insights into the electrical connections of the heart by radiological imaging of the pacing leads capturing the conduction system at various levels ranging from His bundle to individual fascicles**.**

## His bundle pacing

In this study, we found that for reliable His bundle capture, the lead had to be positioned closer to the junction of the membranous and muscular septum. This anatomical location was found to be in RA in most patients (79.4%). Additionally, 87.5% of His bundle capture occurred via RA and only 12.5% showed HBP via RV. Moreover, the patients with HBP via RA had their pacing lead within 2 mm of this anatomical landmark of the junction between the membranous and muscular septum with excellent thresholds at the implantation and follow-up (Table 2). The leads placed more than 2 mm away from this anatomical landmark had higher thresholds at the implantation as well as on follow-up and required lead repositioning.

Furthermore, we observed that to capture His bundle, the lead had to be placed anteriorly towards the TV. The leads placed posterior in the AV septum near NCC of the aorta could not capture His bundle selectively (Fig. 5A). Considering the anticipated location of His bundle anteriorly in the AV septum along the lower edge of the membranous septum, it was not surprising that the pacing leads positioned away from this area had higher thresholds at the implantation or even failed to capture His bundle selectively. Although the higher thresholds were traditionally believed to be due to better insulation of His bundles, we would like to suggest that the distance from the conduction axis could be an equally important contributing factor. This resulted in higher outputs for His bundle capture. All our patients who had the leads within 2 mm of the anatomical landmark of the membranous and muscular septum junction had demonstrated selective His bundle capture at low thresholds during the implantation as well as on follow-up.

A recently published study has reported that in 51 patients with HBP, 11 leads (22%) were placed in the atrial aspect of the His bundle region (36% selective HBP), and 40 leads (78%) were placed in the ventricular aspect (28% selective HBP) [15]. However, in this study TV plane of 2 mm width was made using software and the patients were grouped into those with the leads in the atrial side and ventricular side using this reference. Further delineation of leads was based on complex 3D and 2D plots. We believe that our analysis based on the septal tricuspid valve insertion as the reference point is more anatomical and reliable to assess the lead tip location. In addition to the septal TV insertion, the junction between the membranous and muscular septum could be clearly visualised in our images.

The current study showed that the ventricular component of the membranous septum was seen in only 21% of individuals. The reason for most patients in our study had His bundle capture via RA in contrast to the above study [15] could also be due to the fact that in our patients, almost 79% had no ventricular portion of the membranous septum. This resulted in the junction between the membranous and muscular septum-the anticipated location of His bundle-in RA than RV in most patients in our cohort. This further supported the localisation of His bundle to the anterior AV septum than the interventricular septum. In addition, we could demonstrate the ‘atrial component’ of the muscular septum in one patient which was a novel finding. Since the lead is not normally screwed deeply in HBP, the thick ‘atrial’ muscular septum might have led to the high threshold to capture the conduction system.

## Left bundle branch pacing

The anatomy of LBB markedly varies among individuals and is significantly influenced by the anatomical relationship of His bundle to the interventricular septum [14, 16]. Histopathologic investigations showed the presence of three networks being described as a thin and elongated anterior fascicle, a wider posterior fascicle, and a third septal fascicle [17]. The main left bundle extends inferiorly 10 to 15 mm towards the apex in the mid septal area. Even though in most cases the pacing lead penetrates the mid septal area and can excite the main trunk of the left bundle, occasionally the lead may reach the LAF or LPF during the implantation due to cardiac motion as well as due to the variability of the distribution of the conduction fibres.

In contrast to HBP, LBBP offered wider target areas for capture. The recommended location to screw in the lead to capture the left bundle is 10–15 mm anterior and inferior to the His bundle location into the RV in an imaginary line drawn from His bundle to RV apex. The lead tip to the TV distance was found to be 22.8 mm (mean). The entry point of the lead to the septum was difficult to visualise clearly as it varied in different views and artefacts were interfering with the identification of exact sites. Moreover, the direction of penetration of the lead into the septum was not perpendicular to the septum in all cases. Compared to the location of the septal entry of lead, the lead tip was easier to be identified for measurement purposes.

In addition to the classical location of LBB capture, this study demonstrated individual fascicle capture, with a good threshold and successful outcomes. The demonstration of selective capture of LAF and LPF by ECG as well as CT scan showed clearly that they could be captured individually by the screw-in leads.

Apart from the location in the septum, the depth of penetration of the lead into the septum appears a major determinant for LBB capture. It is important to note that LBB lies subendocardial on the left ventricular side of the interventricular septum, and the lead must be sufficiently deep into the septum to capture the left bundle. Of course, there remains a risk of lead perforation into the left ventricular cavity and damage to the left bundle. CT imaging of the patients with LBBP showed close to 70% depth of penetration of the leads into the interventricular septum (Table 1, Fig. 7). To profile the interventricular septum properly, the LAO cranial view appeared to be the best view in CT images compared to the traditional LAO view under the fluoroscopy.

## Deep septal pacing

There was no identifiable pattern of distribution of the target area for deep septal pacing in CT scan. However, the depth of penetration of the lead was found to be less at a mean of 46% which was significantly less than LBBP. This information led us to believe that many of the deep septal pacing leads might have captured the left bundle if they had been screwed in further.

## Limitations of the study

This study was an observational retrospective study at a single centre involving smaller numbers and was not powered to evaluate the hard clinical end points. CT scan was opportunistic by the clinical indication, and this might have introduced the selection bias into the measurements and observations. However, this study is one of the only two studies [15] which attempted to identify the location of the pacing leads in HBP using cardiac CT scan and the first study to date to our knowledge in LBBP/deep septal pacing. Moreover, the study gives insight into the complex anatomy of the AV septum and its relationship with the conduction system.

## Conclusions

Pacing the anterior part of the AV septum at the junction between the membranous and muscular septum appeared to be the best target to capture His bundle. Lead positioning away from this anatomical landmark might affect the thresholds and long-term performance of HBP. CT scans revealed that most patients had HBP via RA than RV and close to 80% of patients did not have intra-ventricular portion of the membranous septum. This explained the capture of His bundle via RA in most patients. Para-Hisian capture was possible through both RA and RV; however, the leads were seen away from the anticipated location of His bundle. Unlike HBP, LBBP was possible at a wide area in the septum and the selective capture of individual fascicle was feasible. Adequate depth of penetration of the lead was very important to capture the left bundle.

## Supplementary Information

Below is the link to the electronic supplementary material.

Supplementary file1 (DOCX 2926 KB)

## Abbreviations

LBBP: Left bundle branch pacing
LBB: Left bundle branch
HBP: His bundle pacing
RV: Right ventricle
AV: Atrio-ventricular
LV: Left ventricle
RA: Right atrium
RCC: Right coronary cusp
LCC: Left coronary cusp
LAF: Left anterior fascicle
LPF: Left posterior fascicle
ECG: Electrocardiogram
