# Supplementary material for: Computerized tomography image correlation of His bundle/deep septal pacing location and outcomes: an analysis from the Canberra HIs bundle/deep septal Pacing Study (CHIPS)
Source: J Interv Card Electrophysiol. 2022 Jan 27;64(1):137–48. doi: 10.1007/s10840-022-01133-z (PMC9236978; doi:10.1007/s10840-022-01133-z)

**Supplementary Appendix 1**

**Procedure details and selection of study groups**

His bundle mapping was done with Select Secure 3830 lead (Medtronic, Minneapolis, MN) through C315 His fixed curve delivery sheath. The sheath is delivered across the tricuspid annulus, and the His-bundle region is mapped from the ventricular to the atrial aspect with counter-clockwise rotation and withdrawal of the sheath body. The aim is a region with a clear His potential and an appropriate R-wave to p-wave amplitude (generally 3:1). Mapping is performed using standard fluoroscopic views, particularly in the left anterior oblique view to ensure that the lead is opposed to the septal surface of the heart and recording of a His bundle electrocardiogram was attempted in all the cases. If His bundle potential was difficult to record or a His capture was not obtained at the fluoroscopic His bundle site, lead was screwed in at a location where paced surface ECG showed positive QRS in lead II and negative QRS in lead III suggesting para Hisian location.

Selective His bundle pacing was defined by ventricular activation occurring solely over the His-Purkinje system and was recognized by the following criteria: (1) His-Purkinje mediated cardiac activation and repolarization, as evidenced by ECG concordance of QRS and T wave complexes similar to baseline (2) The paced-ventricular interval is almost identical to the His-ventricular interval.

Non-selective His bundle pacing was defined based on capture of basal ventricular septum in addition to His bundle capture as: (1) No isoelectric interval between pacing stimulus and QRS (2) Recording His bundle electrogram on the pacing lead (3) Electrical axis of the paced QRS concordant with the electrical axis of the spontaneous QRS (4) Narrowing of QRS at higher output due to fusion between RV and His bundle capture and widening of QRS at lower output due to loss of His bundle capture or vice-versa

If attempts for His bundle pacing or para-Hisian pacing were not successful, left bundle branch pacing was done. The sheath was turned clockwise and gently advanced 10–15 mm into the ventricle toward the RV basal septum. Unipolar pacing was performed to assess for an ideal site for lead fixation and looked for a paced morphology of QS complex with a notch in the nadir in lead V1 and/or presence of inferior lead and aVR/aVL discordance (R wave in lead II taller than lead III, negative aVR and positive aVL). If the above criteria were met, the lead was fixed to the RV septum by 1–2 rotations and the sheath was gently advanced to abut the septum. Sheath was rotated counter-clockwise to make the sheath tip/lead perpendicular to the septum and further screwing in of the lead was done. Every effort was made to record a left bundle branch potential after screwing in the lead to left bundle branch location.

During implantation, selective LBBP was defined as following: (1) There was an isoelectric line between pacing spike and QRS complex (2) The pacing spike to -QRS duration was same as left bundle potential-QRS interval. During implantation, non-selective LBBP was defined as below: (1) There was no isoelectric line between pacing spike and QRS complex (2) The local ventricular EGM showed direct capture of tissue by the pacing stimulus.

If left bundle branch potential was not recorded or left bundle branch capture could not be obtained after screwing deep into septum despite two or three attempts, deep septal location of the screw in lead was accepted as a bail out strategy.

In this study, 4 different types of pacing were identified based on 12 lead surface electrocardiograms. (Figure 1 A,B,C,D) ECGs were done with pacing out puts kept within physiological range - 1 V at 1ms pulse width to 4 V at 1ms pulse width for His bundle pacing (HBP) and para-Hisian pacing, 1V at 0.4 ms pulse width to 4 V at 0.4 ms pulse width for Left bundle branch pacing ( LBBP) and deep septal pacing.

1. His bundle pacing group: If the 12 lead ECG showed pure His bundle capture at any of the above out puts, such patients are included in HBP group. Capture of His bundle at acceptable output range as described above suggested lead location close to His bundle.
2. Para-Hisian pacing group: Those without a pure His capture at the physiological pacing settings mentioned above, but showing positive paced QRS in lead II and negative QRS in lead III were included in para-Hisian pacing group
3. Left bundle branch pacing group: Selective LBBP was defined in 12 lead ECG at physiological pacing out puts as paced QRS morphology of RBB pattern in V1 with QRS duration less than 120 ms. Non selective LBBP showing qRBB in V1 with QRS less than 130 ms also included in LBBP group. Those patients with LBBP were further analysed for selective fascicular capture as against left bundle branch capture. Those ECGs showing selective conduction system capture (QRS < 120 ms) at physiological pacing out puts as mentioned above, but showing left anterior hemiblock in surface ECG (QRS axis between -45 degrees to -90 degrees, qR complexes in lead I,aVL, rS complexes II,III,aVF) were considered as left posterior fascicle (LPF) capture. Similarly, those with conduction system capture (QRS <120 ms) with left posterior hemi block in ECG (QRS axis 90 degrees to 180 degrees, qR in II,III,aVF, rS in I, aVL) were considered as left anterior fascicle (LAF) capture.
4. Deep septal pacing group: Remaining patients with deep septal pacing who were not showing left bundle branch capture were included in the deep septal pacing group.

**Supplementary Appendix 2**

**Cardiac CT assessment**

All the patients who underwent CT scan of heart in the follow up period were selected for analysis of the location of pacing leads. Best diastolic images were chosen for interpretation. Images were interpreted using Radiant DICOM viewer software (Medixant Maciej Frankiewicz, Poland) initially and subsequently CT images were merged with CARTOSEG CT segmentation module kit (Biosense Webster, Division of Johnson and Johnson CARTO version 6, USA) for detailed 3D image reconstruction. Measurements were done in 3D plane to ensure accuracy and comparability across different patients.

**Anatomical basis for CT measurements**

Kawashima and Sasaki published three distinct patterns of the His bundle observed in 105 human hearts. ([9](#_ENREF_9))(Figure S 1 A, B, C, D). In type I (46.7% of cases), the His bundle coursed along the lower border of the membranous part of the interventricular septum covered with a thin layer of myocardial fibres arising from the muscular part of the septum. In type II (32.4%) the His bundle was away from the lower border of the membranous part of the interventricular septum and was seen within the muscular septum. In type III (21%), the His bundle was ‘naked’ and seen immediately beneath the endocardium and coursed onto the membranous part of the interventricular septum. It is to be noted that all the three types of His bundles described were close to the anatomical landmark of junction between membranous septum and muscular septum.

The second study by Cabrera et al. involving 41 human hearts, described in detail the angiographic, gross macroscopic, and histological dissection of the conduction axis in humans.([10](#_ENREF_10)) Marked variation of the location of the transition from atrioventricular conduction axis to the bundle of His, relative to the landmarks of the triangle of Koch was observed in this study (Figure S1 E,F). In as much as half of both the specimens and the patients, the site of penetration of His bundle was closer to the atrial aspect of the hinge of the septal leaﬂet of the tricuspid valve which was much distal and anterior to the original understanding of “lying within the Koch’s triangle”. In the remaining half of patients, penetrating bundle was observed in the more proximal ‘traditional’ location. Marked variation in the dimensions of the axis and its adjacency to the right-sided endocardium were also found. In about sixty percentage of hearts, an interventricular component of the ﬁbrous membranous septum was not identiﬁed.

These studies provided insights into the location of the His - penetrating bundle system, the former by means of cardiac dissection gave insights into the vertical (superior-inferior) orientation of the conduction system axis with respect to junction of membranous septum and muscular septum and the latter using histology/angiography gave insights into the horizonal (antero-posterior) alignment of the same in relation to septal leaflet of tricuspid valve.([9](#_ENREF_9),[10](#_ENREF_10)) Accordingly, it was assumed that the junction between muscular septum and membranous septum and septal tricuspid insertion point were important landmarks to localise His bundle in cardiac CT.

**CT measurements**

Among the different softwares tried by us, CARTOSEG produced minimum lead artifacts and allowed visualisation and measurements in three-dimensional plane. Of the 37 CTs available for review, three CTs had to be excluded due to poor image quality/lead artifacts. In all remaining 34 CTs, we could identify the lead tip without artifacts in at least one plane. Two investigators did the measurements independently after initial training for lead tip identification and anatomical landmarks. Irregular artefacts and blooming shadows were excluded, and lead tip was identified at the distal most portion consistent with expected shape of lead. Please see Figure S2 given in appendix 2. Red arrows (Figures S2 A,B,C,D) show lead tips clear enough to be acceptable for measurements. Yellow arrows (Figure S 2 C,D) were not considered as lead tips as they were irregular artifacts not consistent with expected shape of lead tip.

By moving images three dimensionally in multiple planes, we could identify proper lead tip in at least one plane in 34 out of 37 CTs reviewed. Once a consensus of interpretability was made between the two investigators, subsequent measurements were made independently, and averages were taken for analysis. It is to be noted that the best plane where a lead tip seen clearly might not be the image plane in which the junction between the membranous and muscular septum was identified and vice versa. Measurements could be made accurately for comparison since in all cases, measurements were done in 3-dimensional planes between clear anatomical landmarks and not in single 2D images.

CT scans of patients in each of the 4 study groups were analysed for the location of the ventricular pacing lead and compared with the existing knowledge on the anatomical landmarks of the conduction system as already discussed. Insertion point of septal leaflet of tricuspid valve and the junction between membranous septum and muscular septum of the atrio-ventricular or interventricular septum were taken as important landmarks to study leads with pure His bundle pacing and para-Hisian pacing. The location in the interventricular septum whether anterior, mid or posterior to capture the respective fascicles were specifically looked for in the case of left bundle branch pacing and deep septal pacing. The following measurements were made, and distances were measured in 3-dimensional plane to offset the effects of cardiac position, shape, and size of patient.

a) Distance between pacing lead tip to the junction between membranous septum and muscular septum in the view best visualized the septum in a patient. LAO Cranial view was found to be the best view to image membranous and muscular part of septum and its relationship with the lead and the septal leaflet of tricuspid valve. (See figure 2). Atrial component and ventricular component of membranous septum was measured in the best view which profiled them.

b) Distance between pacing lead tip to the insertion of septal leaflet of tricuspid valve on to the septum. Location of lead tip in relation to septal leaflet of tricuspid valve and to membranous septum-muscular septum junction was carefully assessed to study the capture of His bundle with respect to RA and RV. Those leads placed distal to septal insertion of tricuspid valve were considered in RV and those placed proximal were considered in RA.

c) Distance between lead tip to lowermost part of non-coronary cusp of aorta (NCC) and right coronary cusp of aorta (RCC). This gave an idea of location of lead in septum in anteroposterior dimension.

d) Location of the lead in interventricular septum in the case of left bundle capture, left anterior fascicular capture and left posterior fascicular capture.

f) Thickness of septum and the depth of lead within septum (This assessment was done when the lead was screwed into muscular part of septum as in LBBP/Deep septal pacing and was not possible at membranous part of septum as in HBP/Para-Hisian pacing)

An intracardiac echo (ICE) image of the anatomical land marks of interest for His bundle capture is shown in Figure S3. Note that the target of His bundle capture is at the junction of membranous and muscular part of septum at the anterior most part of AV septum. Please keep in mind that this image is for representation purpose and not from a study patient; hence, no pacing lead is seen in the image.

**Figure S1: Relevant Anatomy.** Figure **S1A** shows muscular septum and membranous septum in cross section. *Red lines indicate borders of muscular septum and blue lines that of membranous septum. Marked in green is the conduction system (Left bundle branch).* Figures **S1B, 1C** and **1D** show type I, type II and type III His bundles as described.^9^ Note that all the three different type lie near the junction of membranous septum and muscular septum. *Blue lines represent borders of membranous septum, red lines show the border of muscular septum joining with membranous septum, black lines denote attachment of septal leaf leaflet of tricuspid valve (TV). Shaded green is the conduction system. (Compact AV node and penetrating bundle).* Figures **S1E** and **S1F** shows the relationship of conduction system in relation to septal leaflet of TV and Koch’s triangle and its variability as described.^10^ *Blue lines represent attachment of septal leaflet of TV, red lines denote tendon of Todaro and conduction system is shaded in green.* Coronary sinus is seen at the base of triangle. Note the distally and anteriorly placed compact node and penetrating bundle closer to the TV attachment in figure **S1F**.


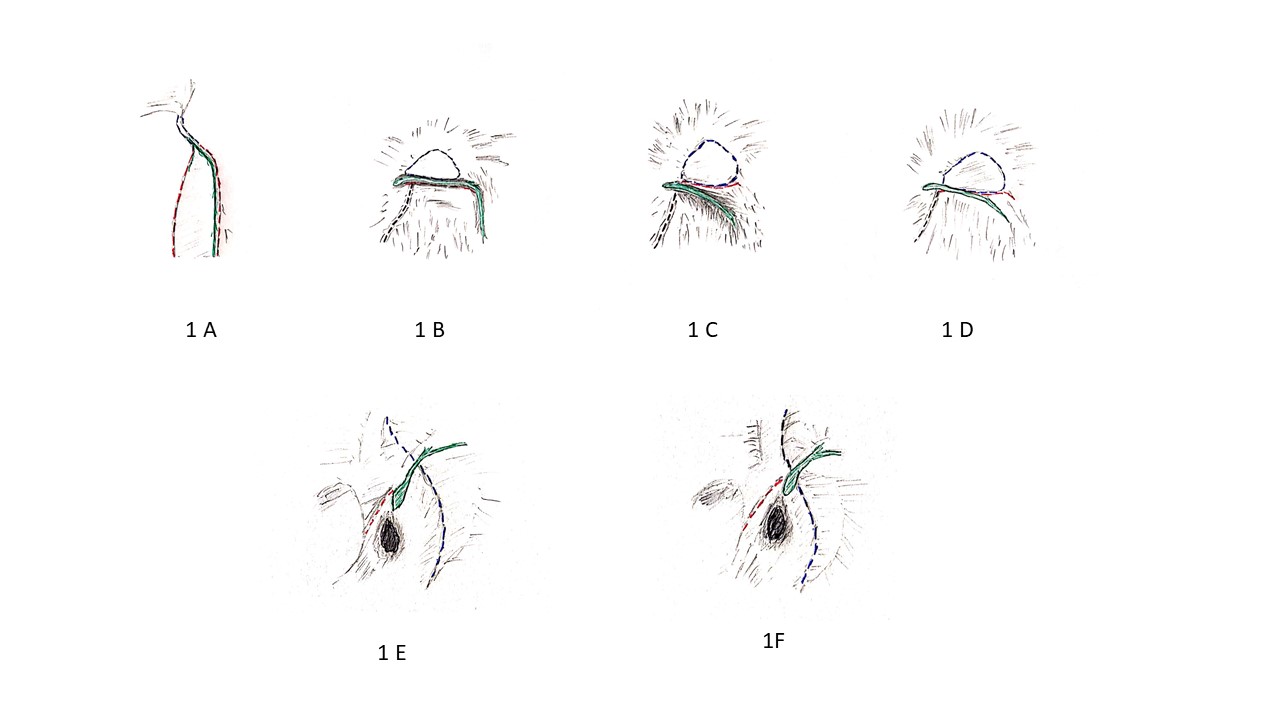


**Figure S2: CT image selection.** Red arrows (Figures **S2 A,B,C,D**) show lead tips clear enough to be acceptable for measurements. Lead artifacts corresponding to yellow arrows (Figures **S2C,D**) were not considered as lead tips as they were irregular artifacts not consistent with expected lead tip shape.


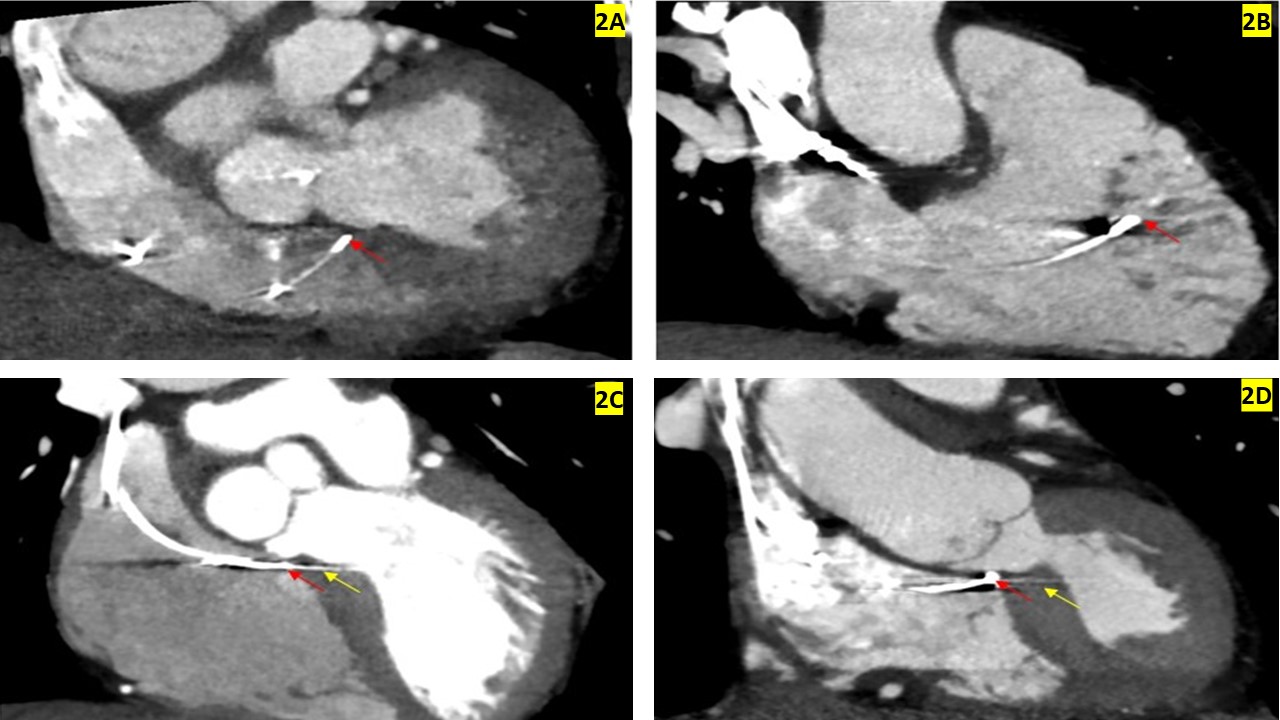


**Figure S3.** Intra cardiac echo (ICE) image showing membranous and muscular parts of septum. Red arrow: Suggested target for His bundle capture. Note that the target is at the junction of membranous and muscular part of septum at the anterior most part of AV septum. Me: membranous septum. Mu: Muscular septum. Ao: Aorta. RA: Right atrium.


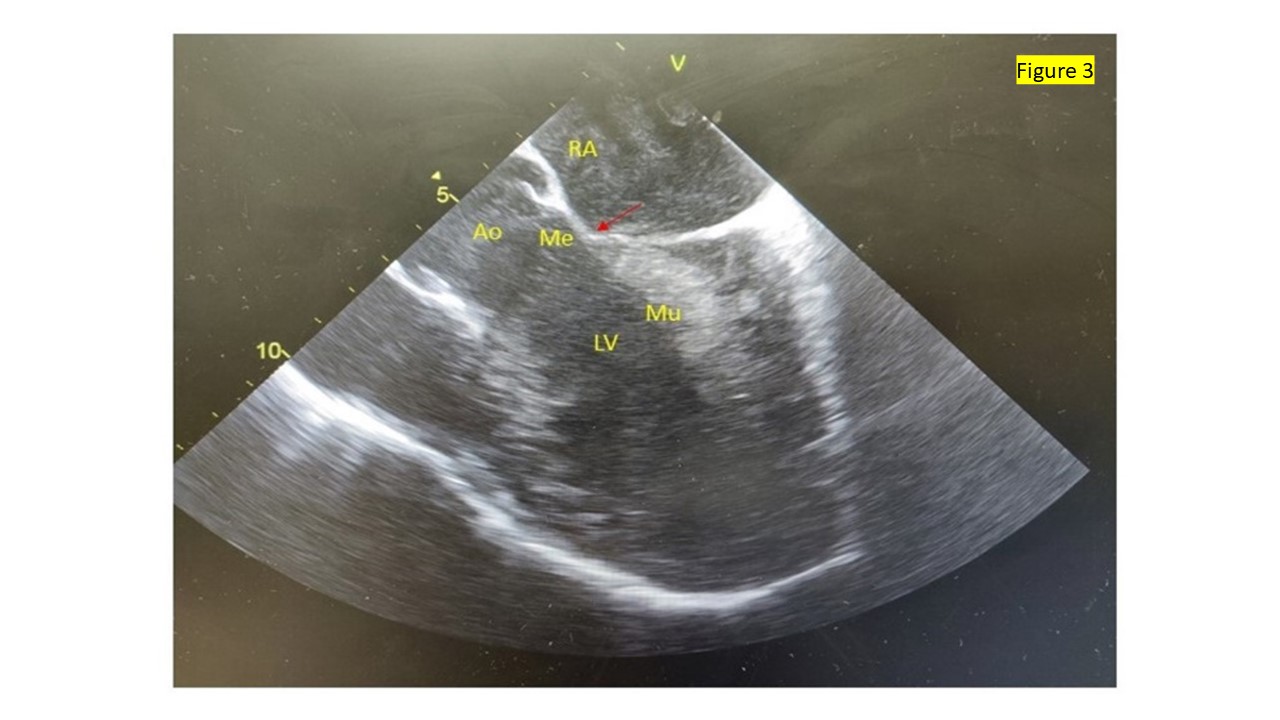


**Supplementary Appendix 3**

**Larger and higher resolution images of figures in the article**

**Figure 2 (A):** His bundle pacing - Note ‘normal looking’ narrow QRS, normal QRS axis and retrograde P waves


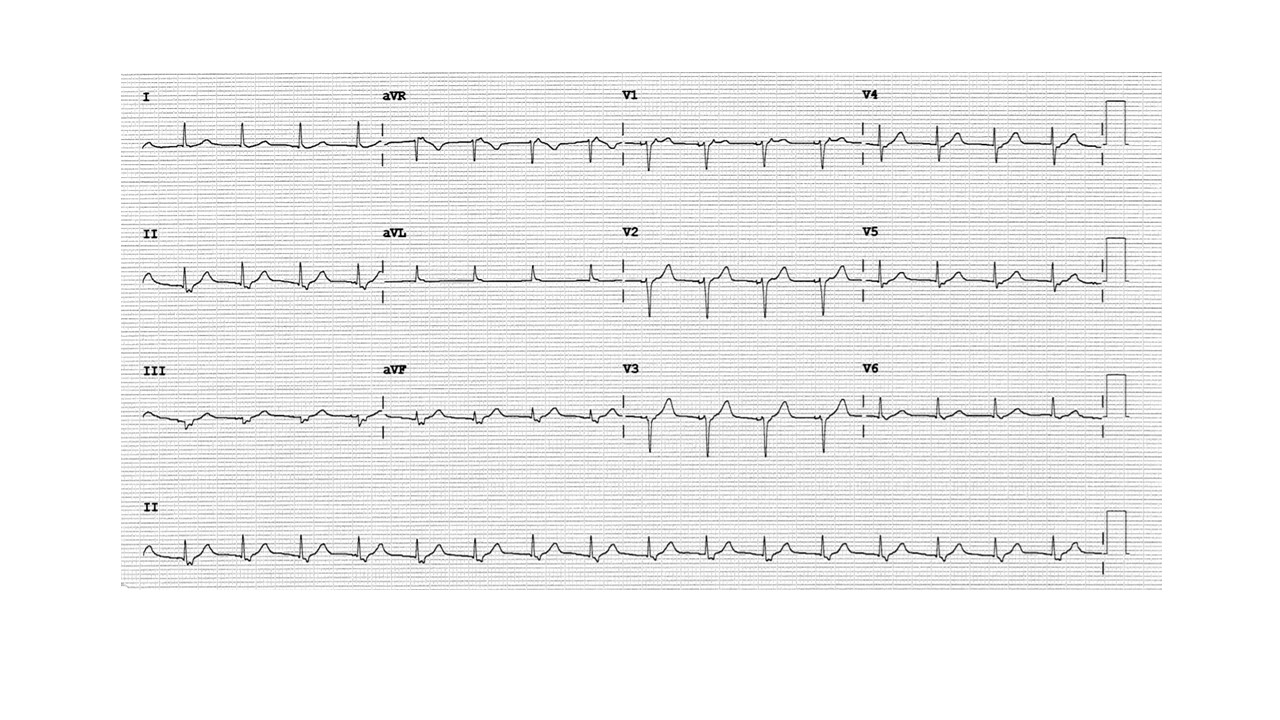


**Figure 2(B):** Para-Hisian pacing - Relatively broader QRS with lead II positive and lead III negative QRS


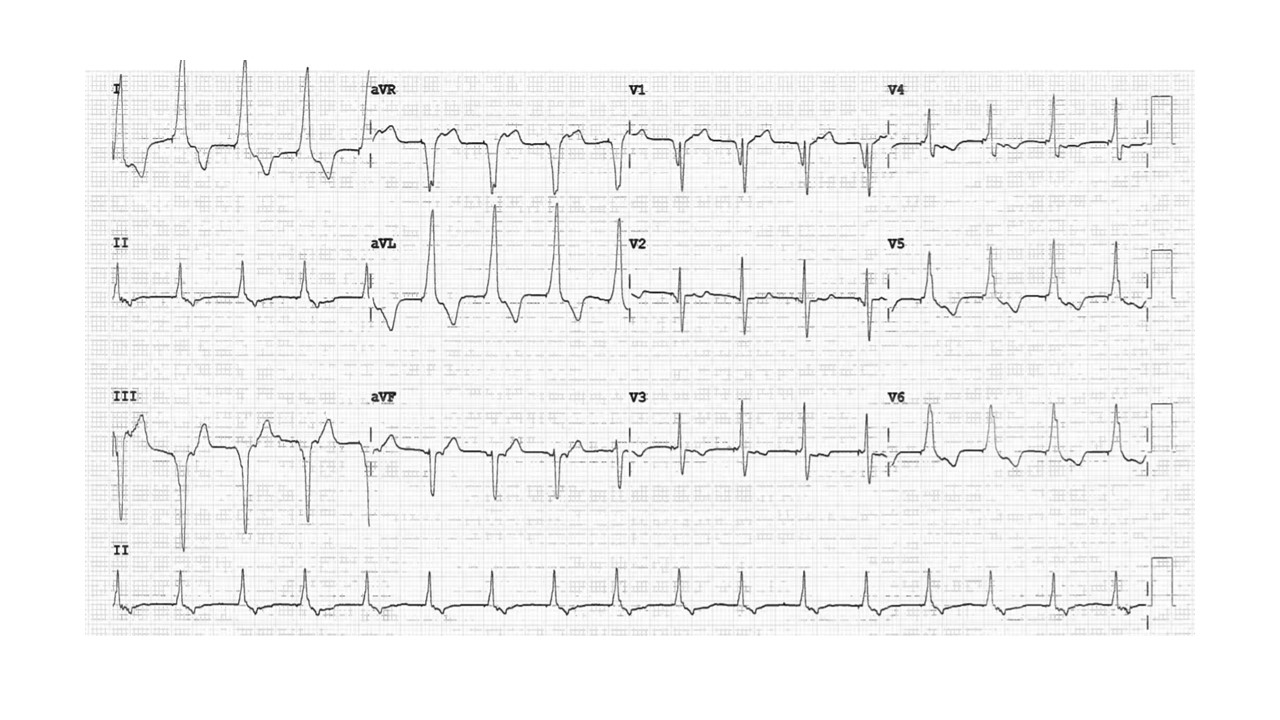


**Figure 2(C):** Left bundle branch pacing - Narrow QRS with qR in V1, normal QRS axis


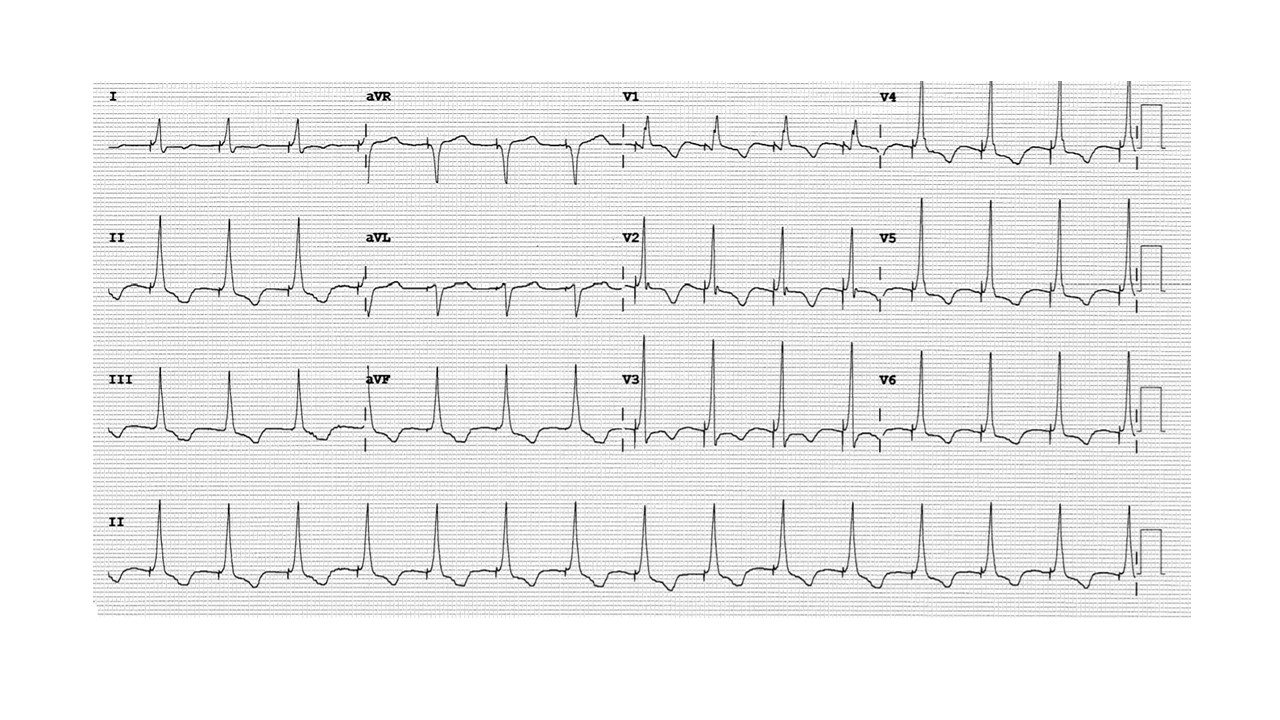


**Figure 2(D):** Deep septal pacing: Note broader QRS and absence of lead II positivity and lead III negativity in comparison to para Hisian pacing.


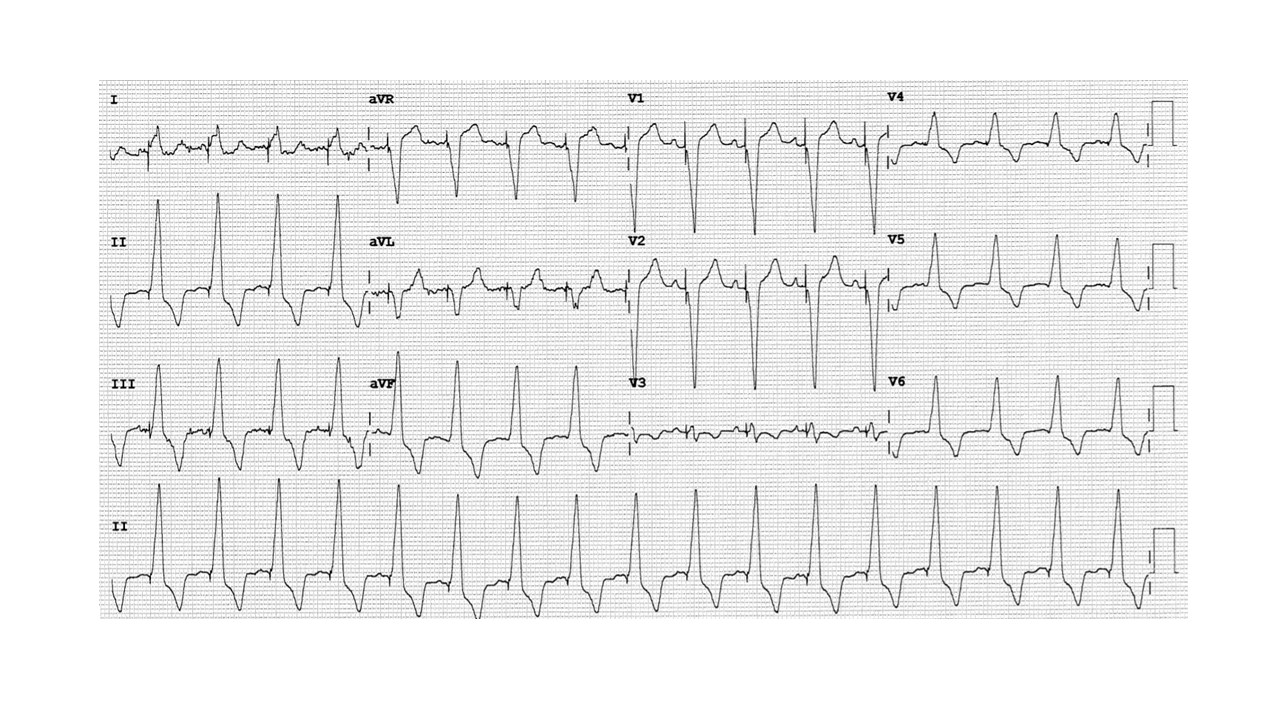


**Figure 3: Relevant anatomy as seen in CT scan.** **3A** Shows various components of septum and relation to septal leaflet of tricuspid valve (STL). Me (Blue arrows): Membranous septum, Mu: Muscular septum, A: Atrial component of membranous septum, V: Ventricular component of membranous septum, Ao:Aorta, Me-Mu (Red arrow) : Junction between membranous and muscular part of septum. Please note that Me-Mu lies below insertion of STL to septum giving rise to ventricular component of membranous septum. Figure **3B** shows absent ventricular portion of membranous septum.


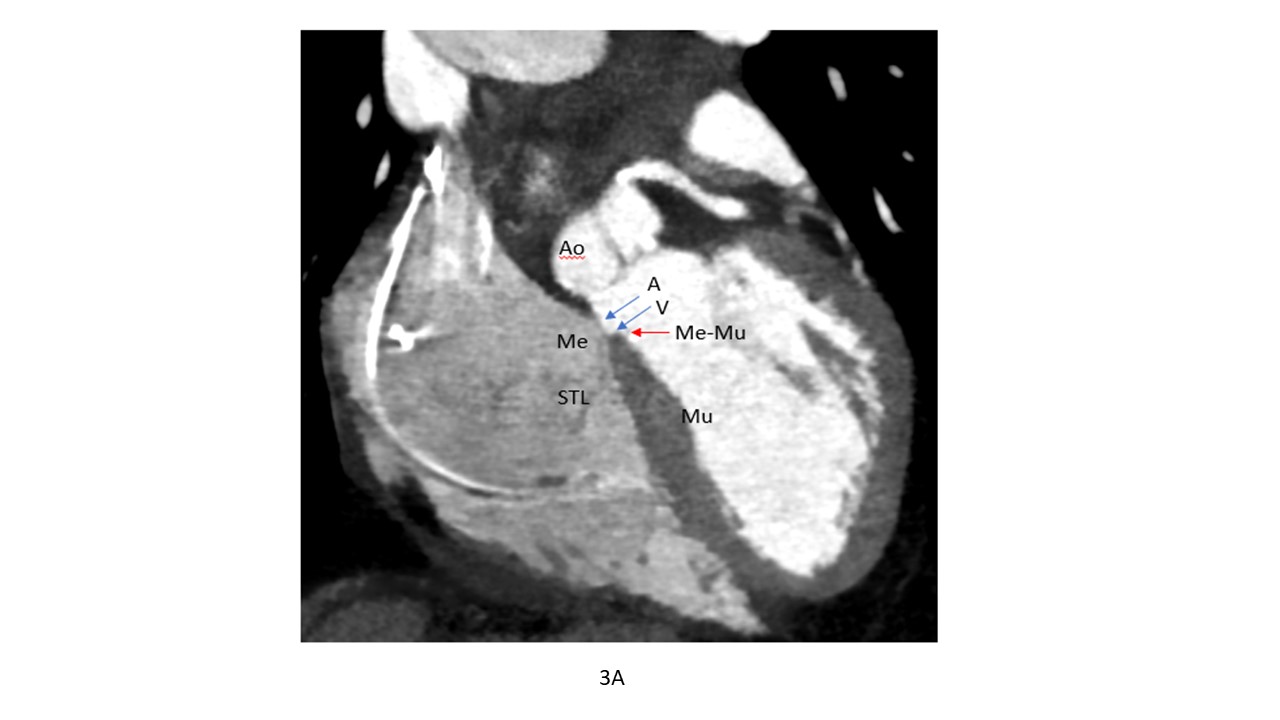


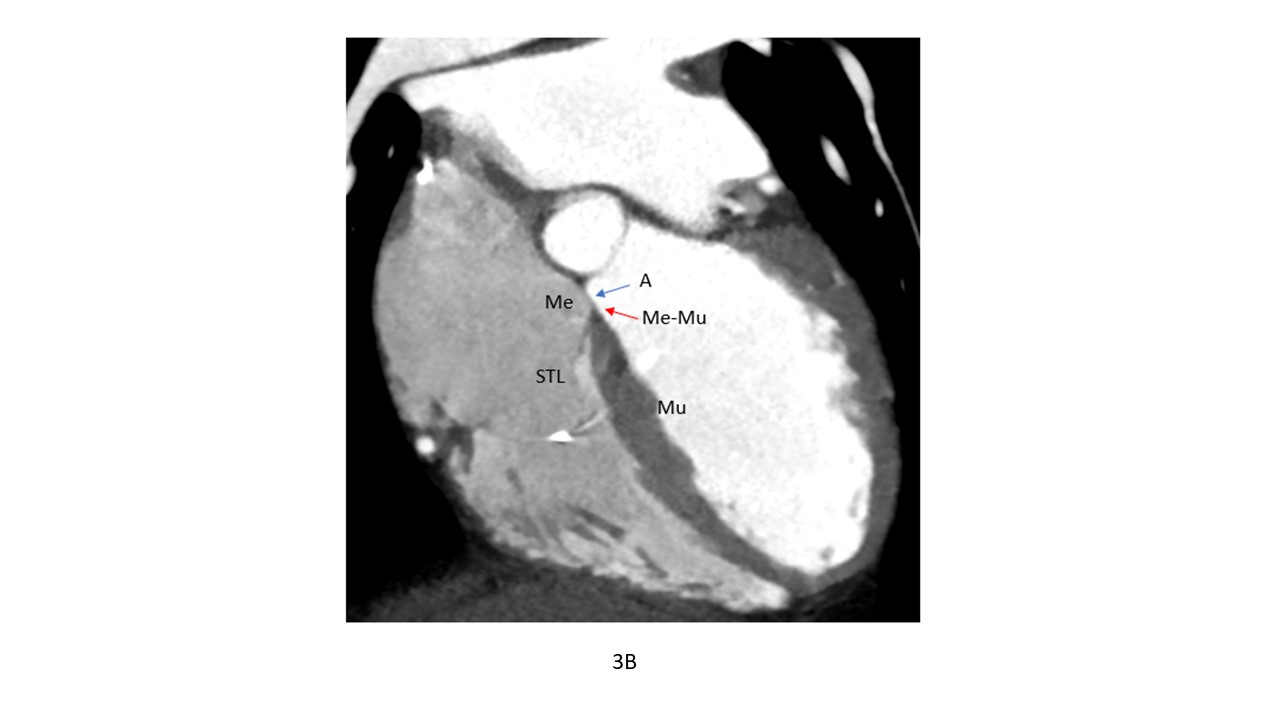


**Figure 4. Location of the lead in His bundle pacing. 4A** shows HBP via RA with lead tip seen anteriorly near right coronary cusp of aortic valve (RCC). Please note lead tip relationship with the Me-Mu junction (red arrow), septal leaflet of tricuspid valve (STL-yellow arrow) and lead (L). Lead tip is seen at Me-Mu junction in this case. **4B** shows HBP via RA who had lead tip beyond 2 mm from Me-Mu junction. This had increased thresholds resulting in lead replacement on follow up. **4C** shows HBP via RV. Lead tip is seen capturing muscular septum distally.


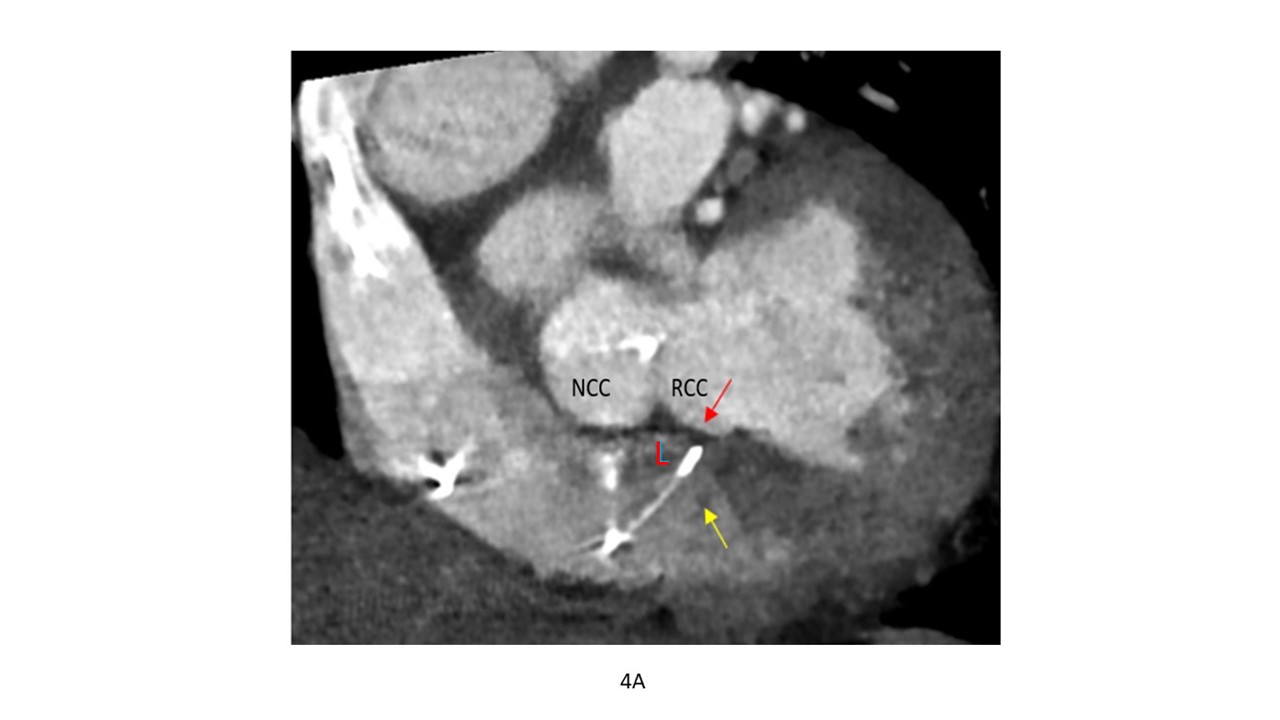


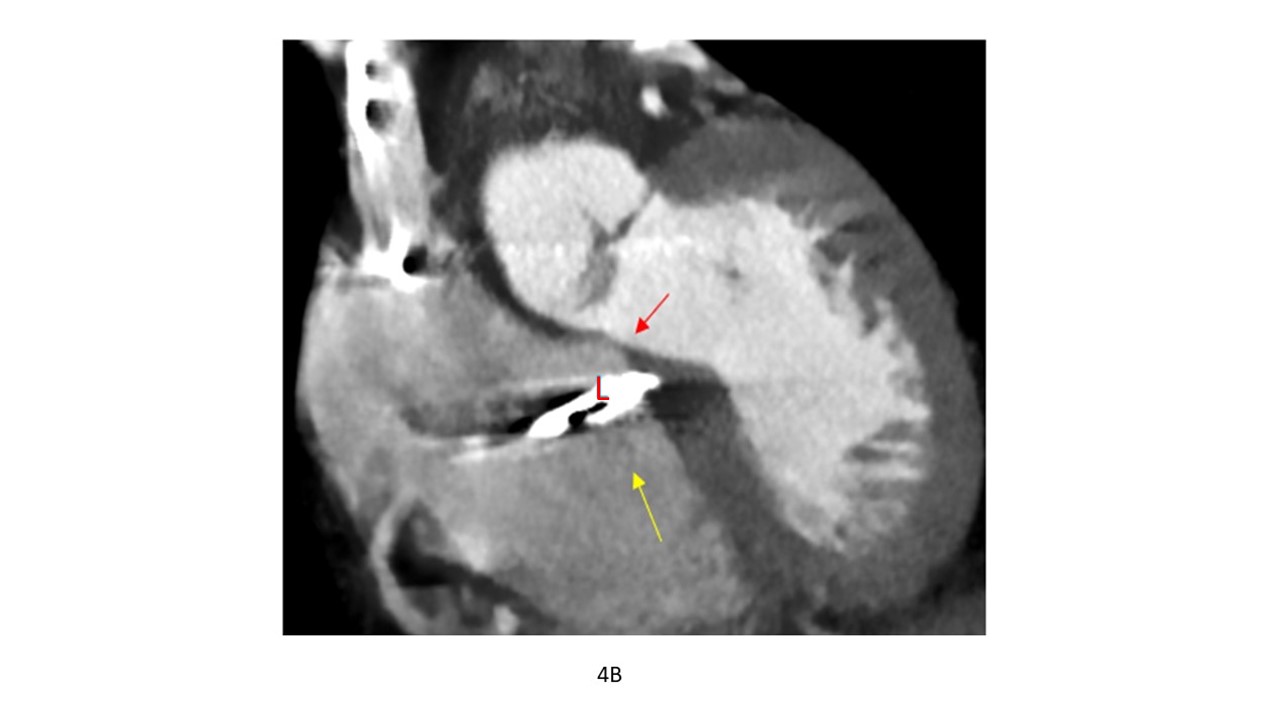


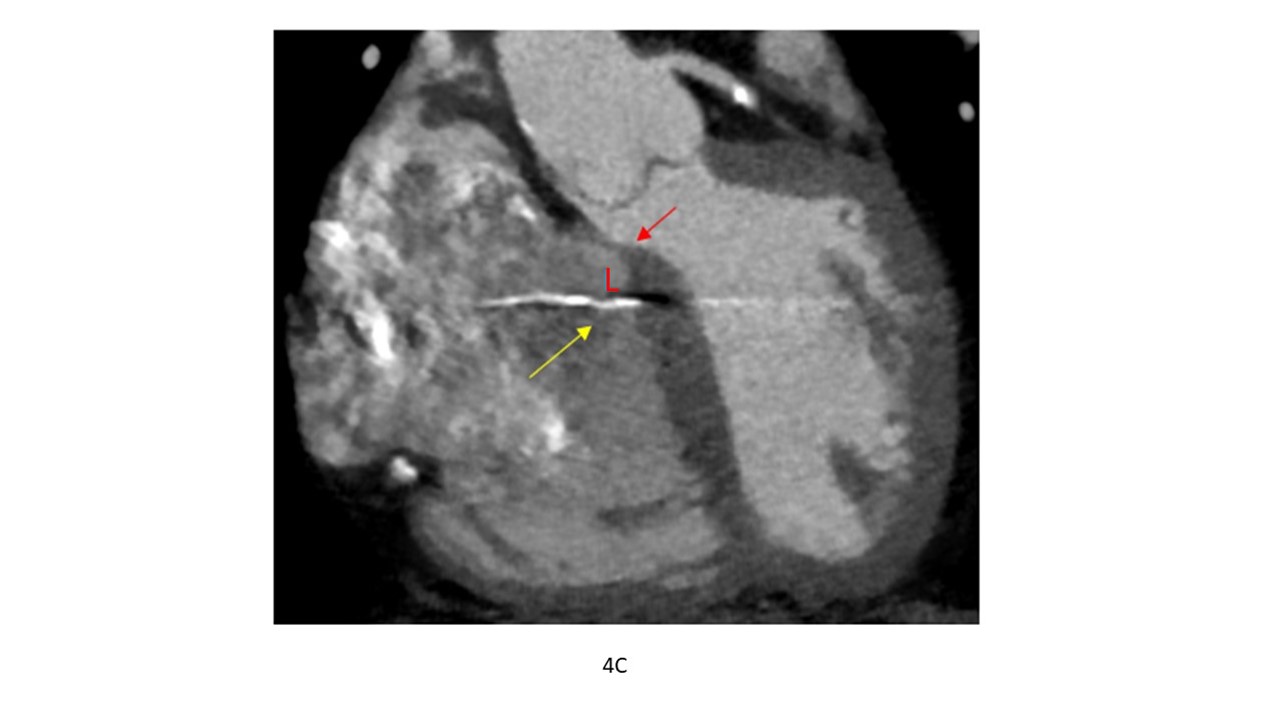


**Figure 5: Location of the lead in para-Hisian pacing. 5A** shows para-Hisian pacing via RA and the lead is seen posteriorly near non coronary cusp of aorta (NCC). Please note that the lead tip is much posterior and away from Me-Mu junction. Me-Mu junction (red arrow), septal leaflet of tricuspid valve (STL -yellow arrow) and lead (L). **5B** shows para-Hisian pacing via RA and the lead tip is located beyond 2 mm from Me-Mu junction. Note ‘atrial component’ of muscular septum. This patient had increased thresholds and required lead revision. Since the lead is not normally screwed deeply in HBP, thick muscular septum might have led to high thresholds to capture conduction system. **5C** shows para-Hisian pacing via RV.


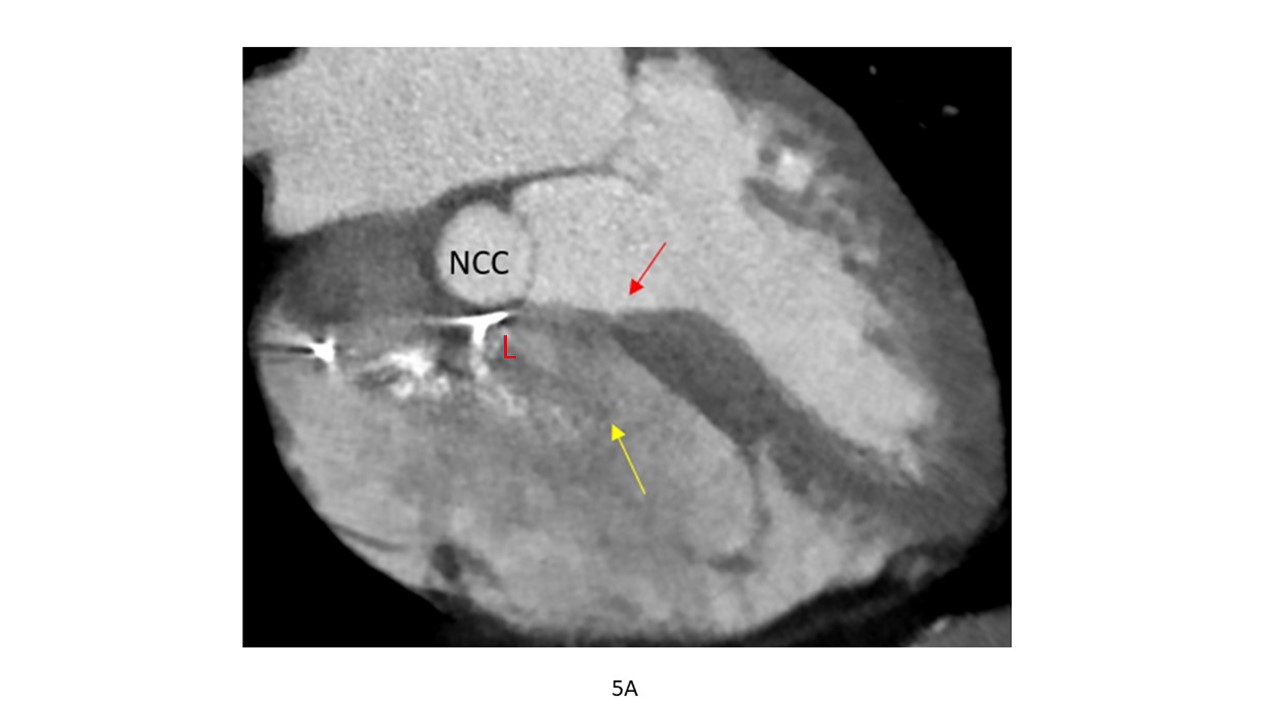


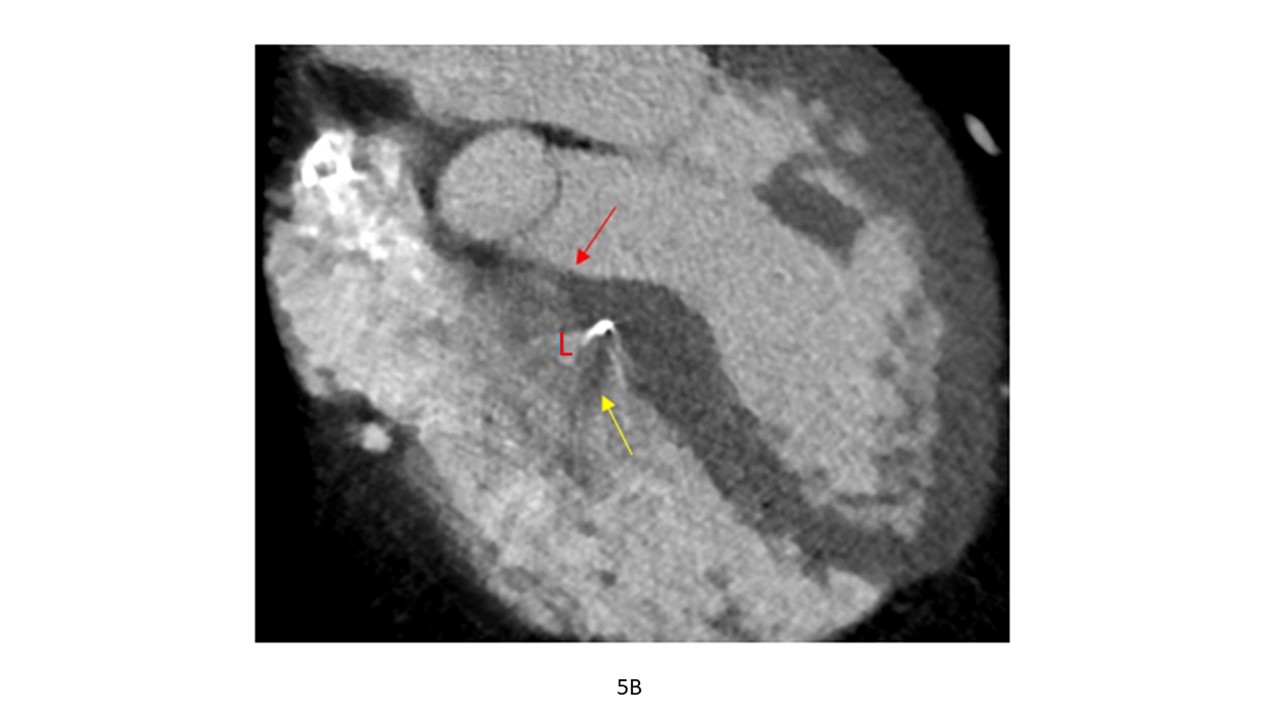


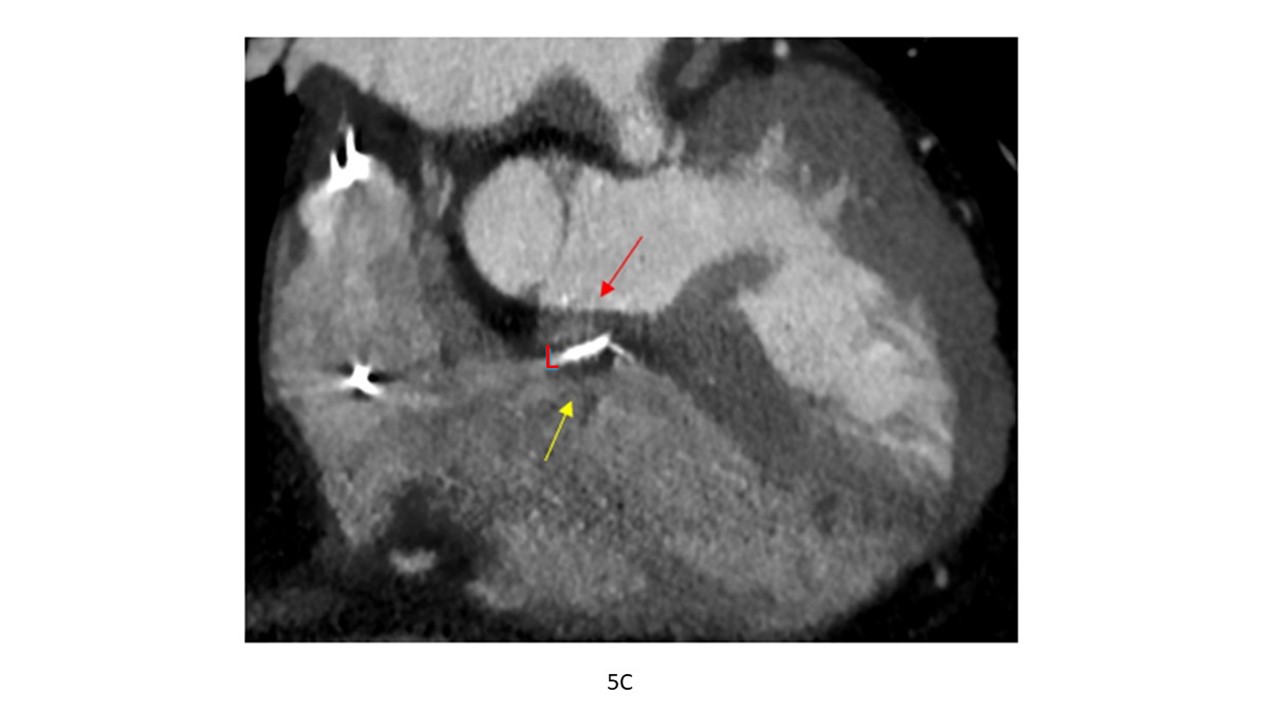


**Figure 6: Left bundle branch pacing** – **6A** shows classical LBBP, **6B** shows left posterior fascicle (LPF) capture, **6C** shows left anterior fascicle (LAF) capture. **6D, 6E** and **6F** shows corresponding CT images of the pacing lead location confirming existing understanding of the location of fascicles of left bundle. Images also confirmed that each fascicle could be individually captured. Red L: pacing lead.


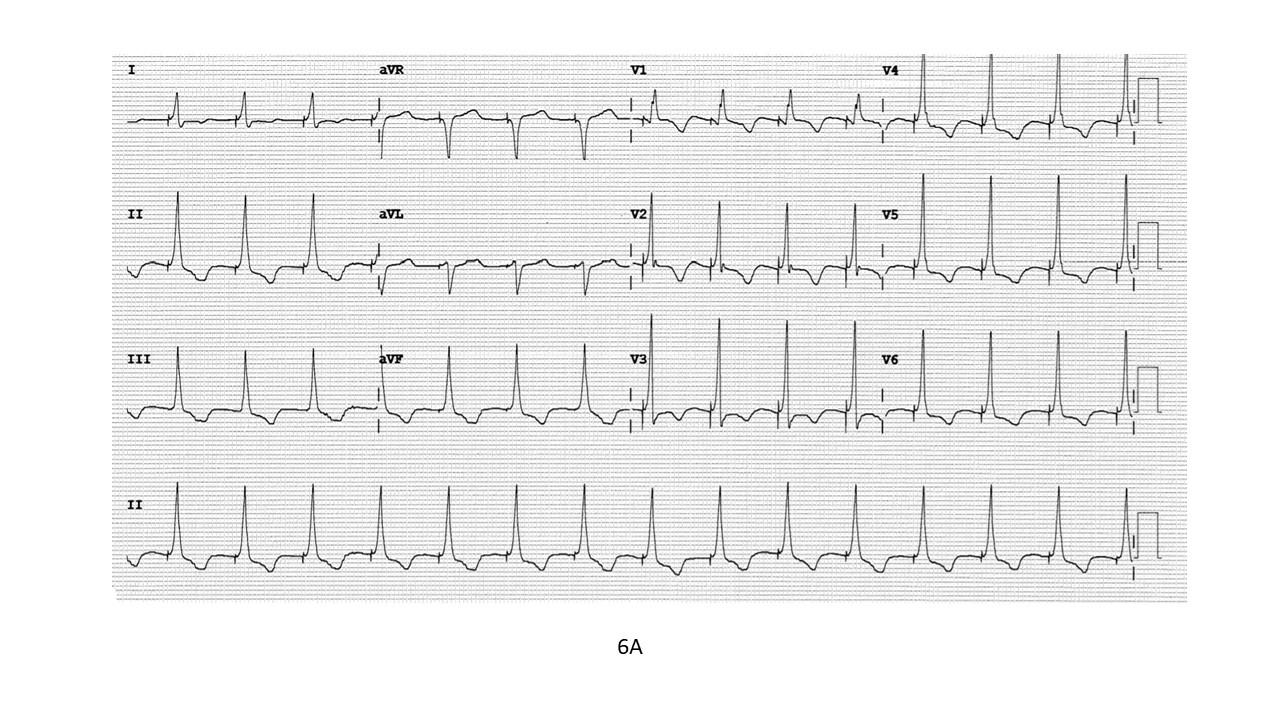


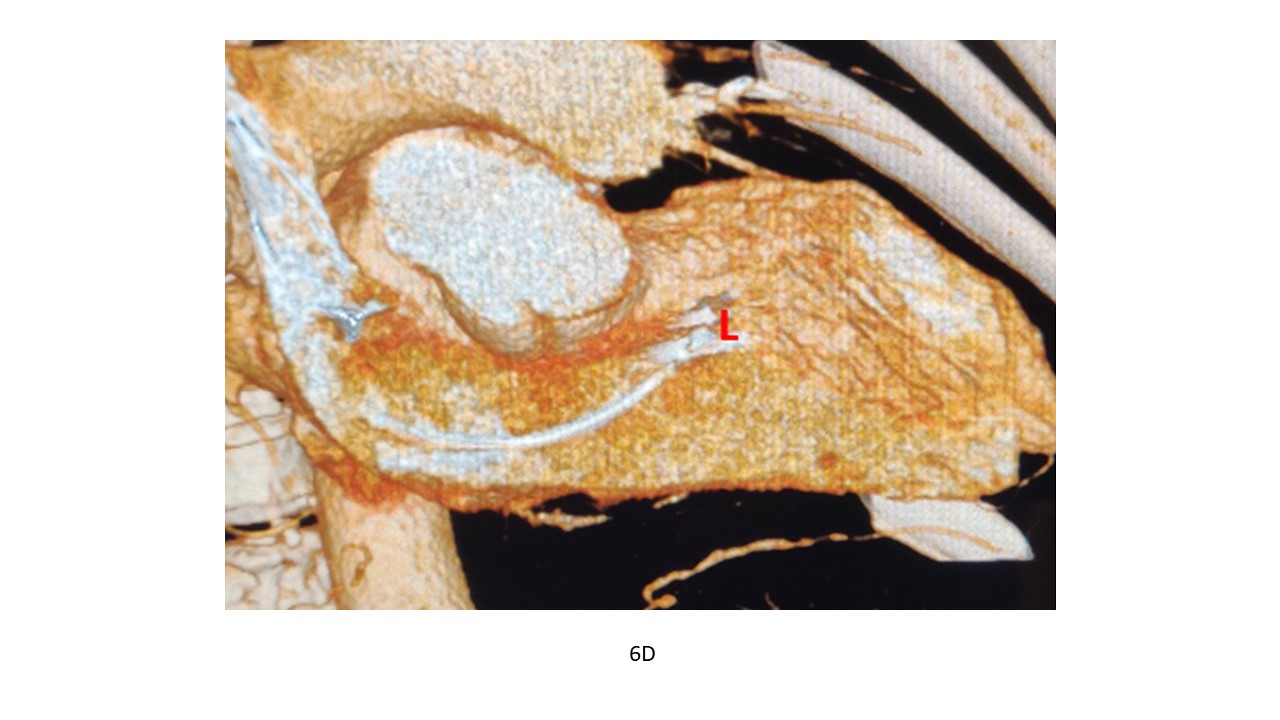


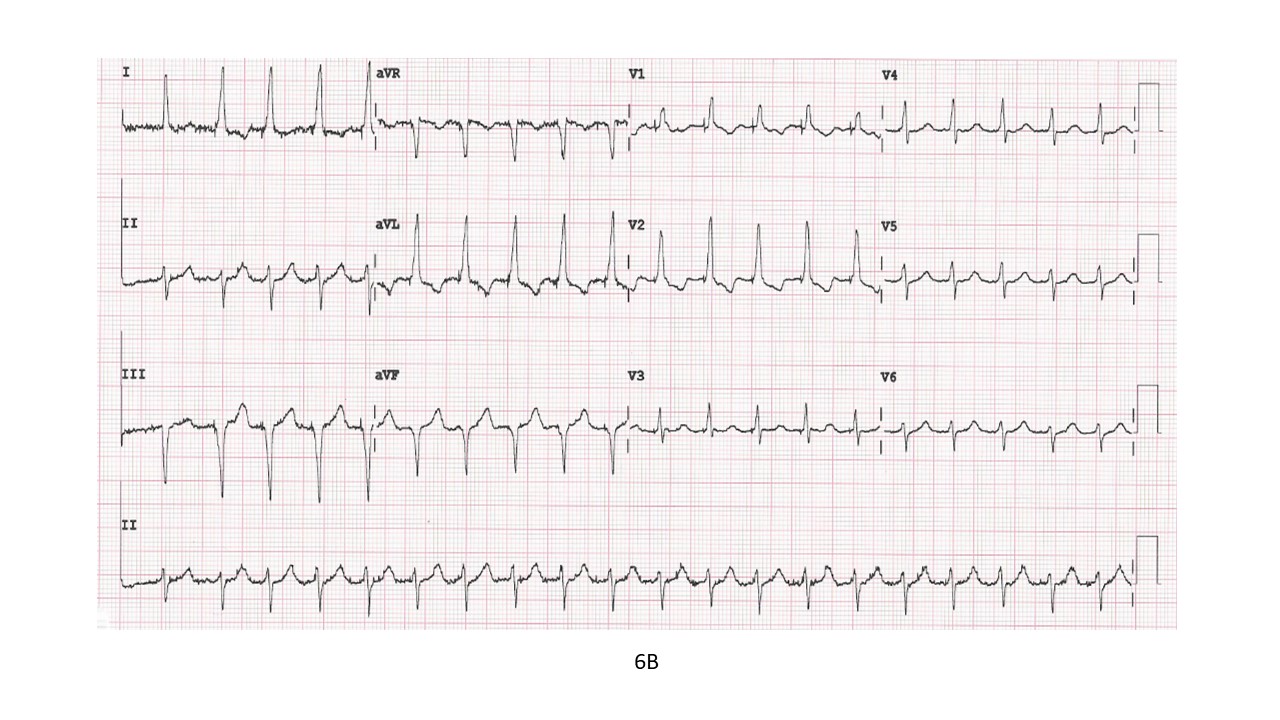


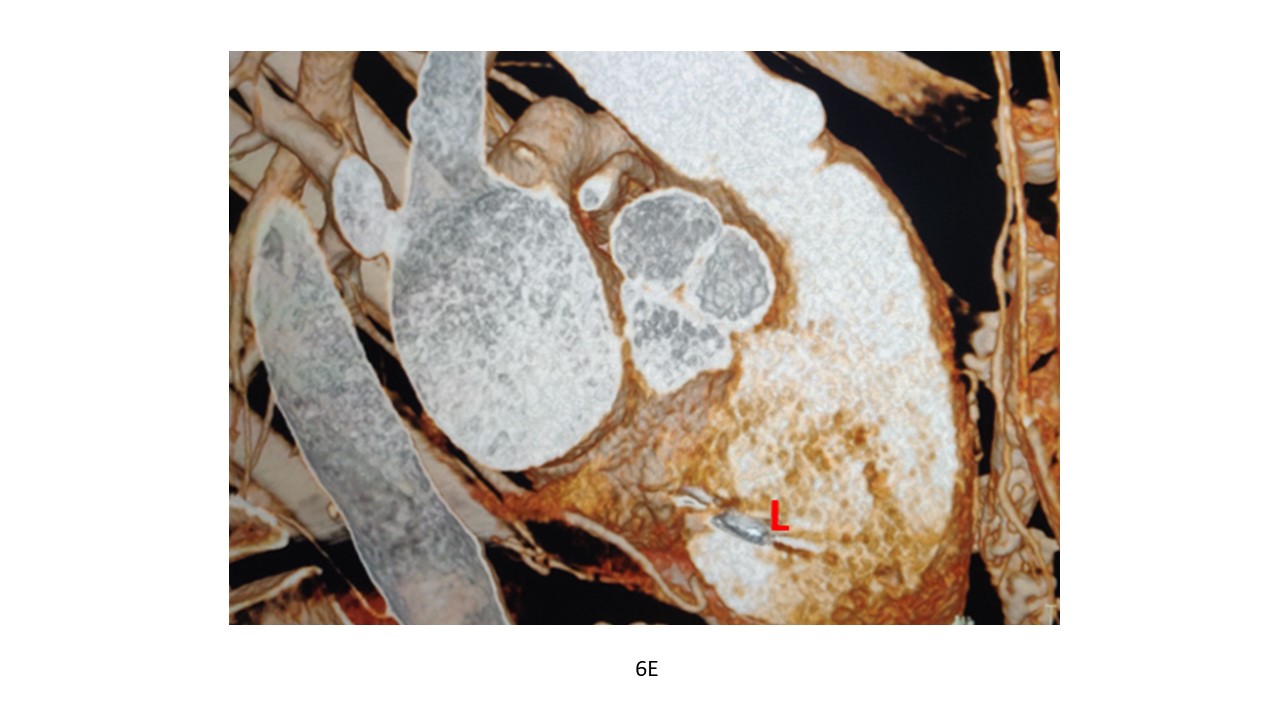


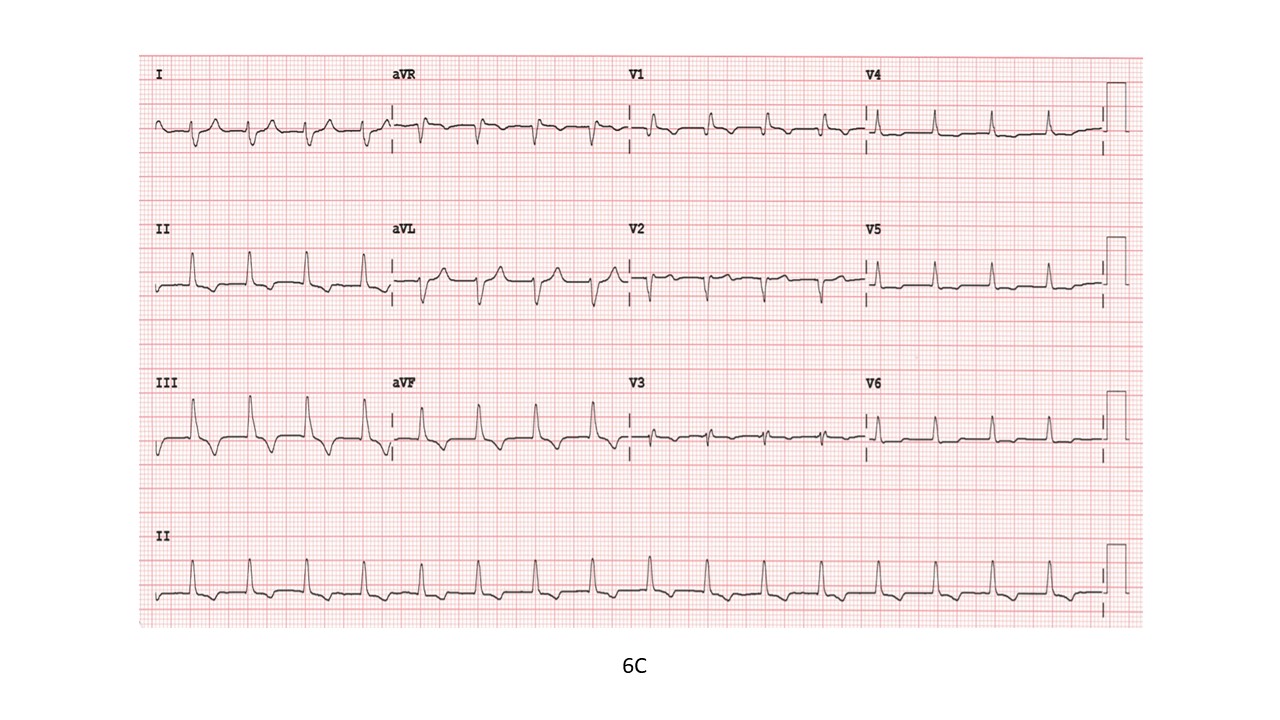


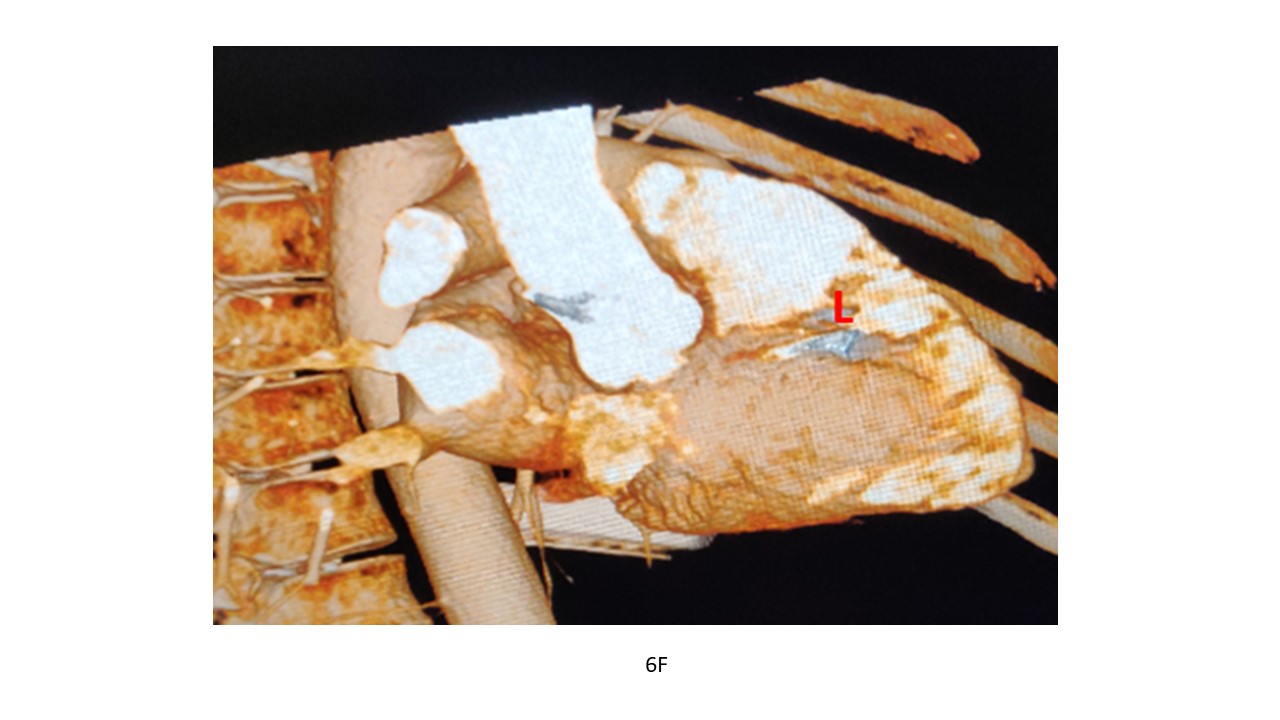


**Figure 7: Depth of penetration of lead into septum.** **7A** shows a case of deep septal pacing. LBBP is shown in **7B.** Deep septal pacing lead has penetrated only up to 30% of depth into septum whereas LBBP lead tip is seen at 80% depth. Red arrow: Lead tip.
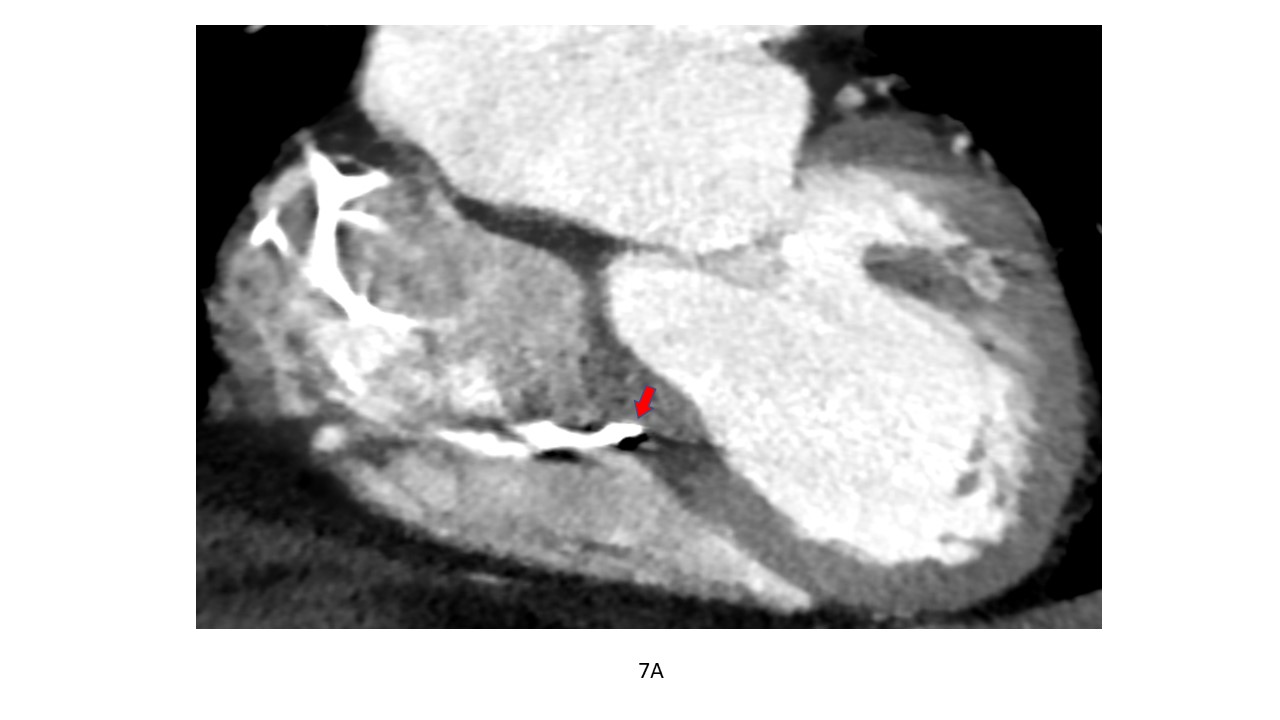

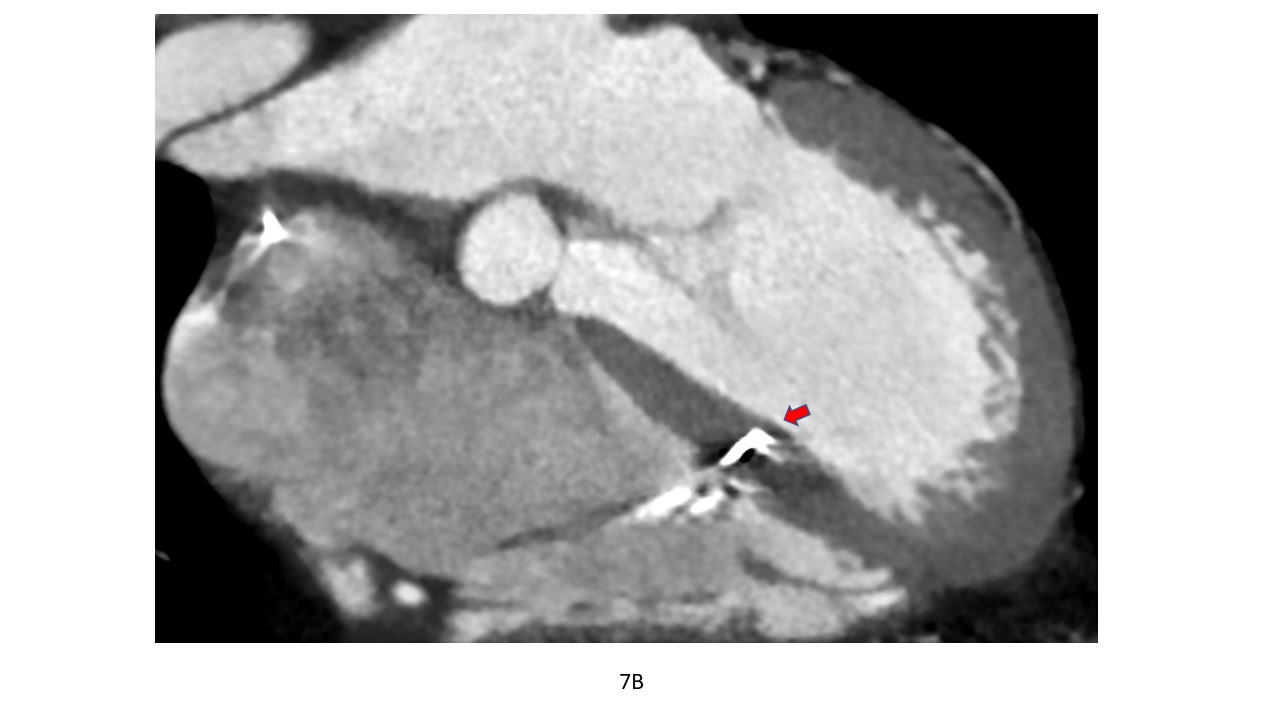

Supplement: Supplementary file 1 — Supplementary file1 (DOCX 2926 KB) [file 10840_2022_1133_MOESM1_ESM.docx]
